# Georeferenced checklist and occurrence dataset of slime moulds (Eumycetozoa) across Central and Eastern Europe emphasising forest ecosystems

**DOI:** 10.3897/BDJ.13.e175486

**Published:** 2025-11-25

**Authors:** Tomasz Pawłowicz

**Affiliations:** 1 Bialystok University of Technology, Bialystok, Poland Bialystok University of Technology Bialystok Poland

**Keywords:** slime moulds, forest ecosystems, Central Europe, Eastern Europe, environmental covariates, substrate, habitat pressure, georeferenced occurrences

## Abstract

**Background:**

A continental-scale, georeferenced checklist of slime moulds (Eumycetozoa) for Central and Eastern Europe, supplemented with standardised environmental covariates and with a particular emphasis on forest ecosystems, has not previously been available. The absence of a harmonised corpus has constrained statistically supported tests of habitat- and substrate-related patterns and limited objective gap-mapping, particularly within forest ecosystems, where microclimatic buffering, dead-wood continuity and stand history are expected to be decisive; it has also hindered rigorous evaluation of slime moulds’ role as bioindicators of forest habitat types, substrate associations and gradients in anthropogenic pressure (naturalness).

**New information:**

Literature discovery spanned multidisciplinary and domain-specific platforms; inclusion required a determinable taxon, a locality at least to country level and a year. Records were de-duplicated conservatively; names were harmonised to a single authority (Eumycetozoa.com) with GBIF Species backbone as a fallback and higher taxonomy was filled consistently. The resource comprises presence-only occurrences, a taxonomically standardised checklist and a reference set; the curated bibliography comprises 528 bibliographic entries. Coverage spans Austria, Belarus, Czechia, Estonia, Germany, Hungary, Latvia, Liechtenstein, Lithuania, Moldova, Poland, Russia (European part), Slovakia, Slovenia, Switzerland and Ukraine. Event dates range from 1857 to 2025-08-01 and support ranges and mixed precision. Environmental content includes elevation, consolidated forest class, substrate category, habitat pressure, microhabitat, pH, air temperature, annual precipitation and stand age; controlled vocabularies comprise eight consolidated forest classes, ten substrate categories and seven habitat-pressure classes. The dataset is released under CC-BY-4.0 (Creative Commons Attribution 4.0 International), employs reproducible DwC mapping and stable identifier versioning and is suited to ecological and biogeographic analyses, including forest-focused modelling and gap analyses.

## Introduction

Slime moulds (Eumycetozoa) are amoebozoan protists that produce fruiting bodies and occupy diverse terrestrial microhabitats, especially in temperate forests (Adl et al. 2012, Leontyev et al. 2019, Stephenson 2023). They exploit moisture‑buffered microhabitats, such as coarse woody debris, bryophyte mats and leaf litter, where they ingest bacteria and other microorganisms and contribute to nutrient turnover (Stephenson 2011, Fukasawa et al. 2015, Leontyev and Schnittler 2017). Phenotypic plasticity — including transitions between flagellated swarm cells and myxamoebae and the formation of dormant microcysts — facilitates persistence under hydric variability in forest environments (Liu et al. 2013, Ing and Stephenson 2022). The dataset and checklist adopt the GBIF‑aligned framework — phylum Eumycetozoa (Mycetozoa) (dataset fields aligned to GBIF; narrative usage: Eumycetozoa) — with classes Dictyosteliomycetes (Dictyostelia), Myxomycetes (Myxogastrea) and Protosteliomycetes (Protosporangiida) and name usage standardised accordingly (Lado 2019, Leontyev et al. 2019).

To date, a continent-scale synthesis for Central and Eastern Europe has not been available, precluding robust tests of habitat- and substrate-related patterns and obscuring spatial gaps that warrant targeted sampling. Forests are a primary venue for slime-mould diversity, where moisture buffering, dead-wood continuity and canopy structure jointly shape community composition; these traits also underpin their role as bioindicators of habitat type, substrate associations and gradients in anthropogenic pressure (naturalness) (Stephenson 2011, Liu et al. 2015, Fukasawa et al. 2015, Leontyev and Schnittler 2017, de Basanta and Lado 2025). A dataset that unifies taxonomy, georeferencing practice and environmental descriptors across countries furnishes the comparability needed for cross‑regional analyses and for forecasting responses to climate and land‑use change. Standardised covariates — elevation, precipitation, pH, stand age, substrate category, consolidated forest class and habitat pressure — enable reproducible tests of hypothesised drivers, while maintaining strict traceability to the original sources. By documenting georeferencing policies (± 10 km assignability, WGS84, explicit uncertainty–precision coupling) and providing full bibliographic provenance, the resource enables reproducible, region‑wide analyses and evidence‑based identification of under‑sampled habitats and areas for targeted surveys.

## General description

### Purpose

To provide a regional, forest‑focused evidence base for slime moulds (Eumycetozoa) in Central and Eastern Europe that supports cross‑study comparability, presence‑only modelling and statistically robust tests of environmental drivers (elevation, precipitation, pH, stand age) across consolidated forest‑habitat, substrate and management‑pressure classes. Occurrences are Darwin Core–mapped and paired with standardised, forest‑relevant covariates; taxonomy is harmonised to a GBIF‑aligned usage, while preserving verbatim identifications to maintain historical signal and transparent reconciliation. Coverage spans the three classes Dictyosteliomycetes (Dictyostelia), Myxomycetes (Myxogastrea) and Protosteliomycetes (Protosporangiida).

## Sampling methods

### Study extent

Temporal coverage uses eventDate with mixed precision (single dates and bounded intervals), spanning 1857–2025; where only a publication year existed, it served as a proxy. Spatial coverage is a 16‑country Central‑ and Eastern‑European domain restricted to the European part of Russia (≤ 60° E); coordinates, when present, are in WGS84. Taxonomic scope is Eumycetozoa (Mycetozoa), covering Dictyosteliomycetes, Myxomycetes and Protosteliomycetes. Data mobilisation occurred in 2022–2025 with subsequent GBIF/Darwin Core harmonisation.

### Sampling description

Sources were identified via multidisciplinary and disciplinary bibliographic platforms and publisher portals; non‑Roman scripts were transliterated using ISO 9:1995 (E) for Cyrillic, preferring established English exonyms where available. Culture‑independent detections (eDNA/metabarcoding) were included; all such occurrences correspond to the locality of the original sampled material. For each eligible source, verbatim text was captured for taxonomy, locality (≥ country), year, coordinates/elevation (if given) and habitat descriptors. Inclusion required a determinable taxon, a country‑level (or finer) locality and a year (sampling or publication as proxy). Provenance was encoded via *basisOfRecord* (*HumanObservation* or *MaterialCitation*). Potential duplicates were screened at publication/locality granularity, retaining distinct sites or dates as separate occurrences. Text was normalised (UTF‑8) prior to Darwin Core mapping.

### Quality control

Taxonomy followed a two‑track approach: *verbatimIdentification* preserves printed usage; *scientificName*/*scientificNameAuthorship* provide the accepted form (*nameAccordingTo* fixed), with higher ranks filled consistently and *taxonRank* restricted to genus/species. Geospatial validation enforced valid latitude/longitude ranges and signs, WGS84 datum, positive non‑zero *coordinateUncertaintyInMetres* and discrete *coordinatePrecision* values; *georeferenceVerificationStatus*, *georeferencedBy*, with decisions noted in *georeferenceRemarks*. Temporal normalisation used ISO‑like *eventDate* formats (year, year‑month, full dates, intervals) cross‑checked against year/month/day (ISO 8601), leaving unknown components blank. CSV hygiene ensured fixed column counts, correct quoting/escaping, LF endings and UTF‑8 encoding; numeric fields use dots and plain decimals. Locality handling preserved *verbatimLocality* and harmonised names to English exonyms or consistent transliteration; *stateProvince* was retained as supplied. Country names and *countryCode* were validated as paired fields. Elevation, when present, was duplicated to *minimumElevationInMetres* and *maximumElevationInMetres*.

### Step description

A schematic overview of the occurrence‑data curation workflow from source intake to versioned release is shown in Fig. 6. A detailed, step‑by‑step operational description follows below (steps 1–23) and corresponds directly to the elements depicted in the schematic.

- Step 1. Establish the project namespace and templates; register controlled vocabularies (8 consolidated forest classes, 10 substrate categories, 7 habitat‑pressure classes) and unit/rounding rules for *dynamicProperties* keys.
- Step 2. Discover and screen sources against inclusion criteria; register provisional metadata and eligibility flags.
- Step 3. Acquire full texts; standardise encoding to UTF‑8; create Harvard‑style *associatedReferences* and normalise punctuation and spacing (full bibliography is listed comprehensively in Suppl. material 1).
- Step 4. Record evidence type and de‑duplicate at publication/locality granularity: set *basisOfRecord* (*HumanObservation* or *MaterialCitation*); screen duplicates with the key {authors + year + title + country + verbatimLocality + DOI}.
- Step 5. Extract verbatim taxon strings into *verbatimIdentification*; attribute *identifiedBy* per source (or authors when unspecified); set the authority in *nameAccordingTo*.
- Step 6. Reconcile names to accepted usage: populate *scientificName* and *scientificNameAuthorship*; fill higher ranks with *taxonRank* restricted to “*genus*”/“*species*”.
- Step 7. Parse and normalise dates: write *eventDate* (single date or start/end interval) and parsed components *year*, *month*, *day* (ISO 8601); where sampling date is absent, use publication year (year‑only).
- Step 8. Harmonise countries and codes: populate *country* (chosen lexicon) and *countryCode* (ISO‑3166‑1 alpha‑2); retain provider *stateProvince* without additional normalisation.
- Step 9. Build locality pair: keep exact *verbatimLocality*; generate harmonised *locality* (English exonym where established, otherwise consistent transliteration).
- Step 10. Transcribe supplied coordinates into *decimalLatitude* and *decimalLongitude*; capture *verbatimCoordinates*, *verbatimCoordinateSystem* and *verbatimSRS* when provided; set *geodeticDatum* = WGS84.
- Step 11. Geocode absent/ambiguous sites via authoritative gazetteers and apply the ± 10 km localisation rule; record *georeferencedBy*, *georeferenceVerificationStatus* (“verified”/“requires verification”).
- Step 12. Couple uncertainty to precision: assign non‑zero *coordinateUncertaintyInMetres* and apply the six‑value mapping to *coordinatePrecision* {0.1, 0.01, 0.001, 0.0001, 0.00001, 0.000001}; round *decimalLatitude*/*decimalLongitude* accordingly.
- Step 13. Handle elevation: parse *verbatimElevation* and duplicate single values into *minimumElevationInMetres* and *maximumElevationInMetres*; keep integers in metres.
- Step 14. Map habitat: derive one *consolidatedForestCategory* (8 fixed labels) (Table 3) from source wording; store detailed verbatim stand text in *forestTypeDetailed*.

- Step 15. Map substrate and microhabitat: assign *substrateCategory* (10 fixed labels) (Table 4) and retain free‑text *microhabitat* (substrate, host, decay state, height).

- Step 16. Add environmental covariates to *dynamicProperties* (minified JSON with explicit‑unit keys *airTemperature_C, annualPrecipitation_mmYr, pH, standAge_yr, consolidatedForestCategory, substrateCategory, habitatPressure*) (Tables 3, 4, 5); enforce rounding (temperature 0.1°C; pH 0.01; precipitation and stand age as integers) and normalise source decimal commas in pH to numeric dots.

- Step 17. Populate status and licensing constants: set *occurrenceStatus* = “PRESENT” and licence = “CC‑BY‑4.0”.
- Step 18. Construct stable identifiers: generate a base *occurrenceID* as UUID v5 from a fixed namespace over {DOI (or empty) + authors + year + title + country + verbatimLocality}; exclude coordinates from the key. If multiple records resolve to the same base UUID, append an alphabetical disambiguator (“-a”, “-b”, …) to the later records to guarantee uniqueness while preserving the base UUID.
- Step 19. Core QA — spatial: validate *decimalLatitude* ∈ [−90, 90] and *decimalLongitude* ∈ [−180, 180], hemisphere signs, dot as decimal separator, plain‑decimal coordinatePrecision and positive *coordinateUncertaintyInMeteres*.
- Step 20. Core QA — textual and codes: verify country ↔ *countryCode* pairs; check UTF‑8 normalisation; preserve stateProvince endonyms/transliterations; ensure locality/verbatimLocality rule consistency.
- Step 21. CSV hygiene and export: enforce fixed column counts per row, correct quoting/escaping for commas/quotes/newlines, LF line endings; write Darwin Core Occurrence CSV with all required fields present.
- Step 22. Duplicate audit and consolidation: re‑scan post‑export for residual duplicates; merge only truly redundant records, retaining distinct dates/sites as separate occurrences.
- Step 23. Versioning and release: compile a changelog, stamp dataset version and package metadata; verify uniqueness of *occurrenceID* prior to submission. The dataset is maintained on an irregular, but not less than biennial schedule; update timing is driven by the emergence of new literature and by the acquisition of materials from authors and repositories (including monographs and inventories reporting previously undocumented occurrences) to extend the dataset and remedy documented gaps. Version identifiers follow a Major/Minor/Patch scheme.

## Geographic coverage

### Description

**Figure 1.**
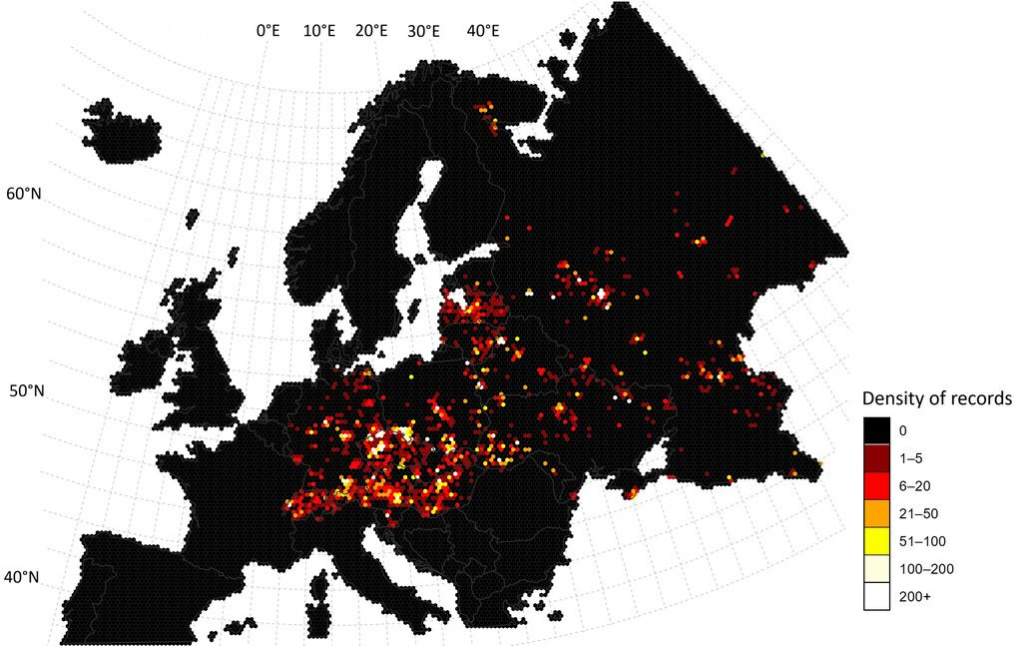
Spatial distribution of Eumycetozoa records aggregated to 25‑km hexagons; colour classes show counts per hexagon (0, 1–5, 6–20, 21–50, 51–100, 100–200, ≥ 200). Coverage spans 16 countries in Central and Eastern Europe, with records limited to the European part of the Russian Federation (≤ 60° E).

The dataset encompasses Central and Eastern Europe as delimited in the project schema, with records encoded at country and paired *countryCode* (ISO 3166‑1 alpha‑2). The represented states are shown in Table 1. Spatial gaps are evident (see Fig. 1).

Locality information is provided in two complementary fields. The harmonised Latin‑script string in *locality* adopts internationally recognisable English toponyms where established or a consistent transliteration otherwise (ISO 9:1995 (E) for Cyrillic). The exact source wording, including language, diacritics and punctuation, is preserved in *verbatimLocality*. Subnational context in *stateProvince* is retained as supplied; heterogeneous endonyms and transliterations (e.g. voivodeships, oblasts, Länder) are not normalised further.

Coordinates are supplied only when a site could be localised to within ± 10 km. In such cases, *decimalLatitude* and *decimalLongitude* are given in decimal degrees with *geodeticDatum* = WGS84, and every coordinate pair is accompanied by explicit *coordinateUncertaintyInMetres* and a matching *coordinatePrecision* (rounding tied to uncertainty). Provenance and adjudication are recorded via *georeferenceVerificationStatus* and *georeferenceRemarks*. Georeferencing was performed using various websites and software tools, such as GeoNames, OpenStreetMap, Wikipedia and Ramsar and, in most cases, multiple sources were used together. Observed coordinate ranges in the delivered dataset are as follows: *decimalLatitude* corresponds to mid‑ to high‑northern latitudes, with examples 41.60–41.83 for Dagestan localities (e.g. Samursky Zakaznik) and 67.76–67.95 for northern sites (Khibiny Mountains; Laplandskiy State Nature Biosphere Reserve). *decimalLongitude* values are all positive and span approximately 6.16°–59.62° E, with illustrative points at 6.16° (Col de Saint‑Cergue) and 59.62° (Visim Nature Reserve). Numerical precision varies (one to three decimals), consistent with heterogeneous source precision and georeferencing, and identical longitudes/latitudes can recur across records at the stated precision.

Spatial coverage is uneven: Poland shows high overall totals, but under‑sampled belts in the north‑west, north and parts of the north‑east; Germany is sparse along the western flank (north‑west and south‑west); Ukraine is largely unsampled in the centre–south and east, with Crimea mostly lacking, except the south coast (recent geopolitical constraints apply); Belarus concentrates near Minsk and Białowieża, while the east, north‑east and south‑east remain thin; in the Baltics, Estonia is especially sparse, Latvia is clustered centrally and Lithuania is comparatively better in the south‑east and along the western coast; Liechtenstein has very few records (collateral to Swiss surveys); Hungary is generally well studied, but thinner in the east; Czechia reaches very high density relative to area with thinner coverage in the east; Moldova has very few records; European Russia features dispersed coverage with foci near Moscow, Tula, Smolensk and Tver and additional work in Kalmykia and Karelia, while vast areas remain sparsely sampled (see Fig. 1).

### Coordinates

40 and 70 Latitude; 10 and 60 Longitude.

## Taxonomic coverage

### Description

The corpus is confined to slime moulds (Eumycetozoa) and includes records from all three classes used in the dataset’s harmonised framework: Dictyosteliomycetes (Dictyostelia), Myxomycetes (Myxogastrea) and Protosteliomycetes (Protosporangiida). Higher ranks are populated in Darwin Core as constant or categorical fields: kingdom = Protozoa, phylum = Mycetozoa (Eumycetozoa) and the full set of *class*, *order*, *family*, *genus*, *specificEpithet*, *infraspecificEpithet*, the binomial species and *taxonRank* restricted to “genus” or “species”. In the harmonised name set, the species‑level component comprises 624 distinct taxa; orders and families are represented across eleven and twenty‑four categories, respectively. A dual‑name strategy preserves the original and accepted usages. *verbatimIdentification* stores the printed string exactly as it appears in the source (including spellings, authorships, qualifiers and infraspecific ranks). *scientificName* and *scientificNameAuthorship* provide the accepted form for indexing and analysis, with higher classification filled consistently. Attribution of the determination is captured in *identifiedBy*; where an identifier was not specified, the publication’s author(s) are recorded per the curation rule.

**Figure 2.**
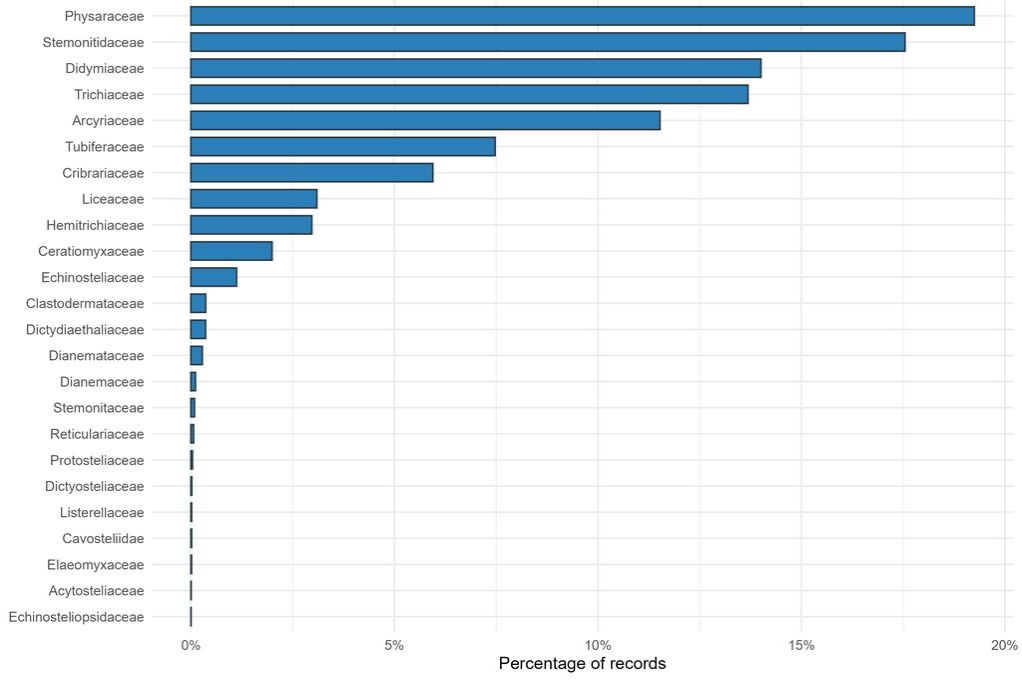
Family composition of Eumycetozoa records is shown as percentages; with counts summarised in the underlying dataset.

The taxonomic backbone is enforced via *nameAccordingTo* = Eumycetozoa.com; where a name was unresolved there, the GBIF Species backbone served as the fallback authority. At the family level, composition is dominated by Physaraceae (n = 7030) and Stemonitidaceae (n = 6408), followed by Didymiaceae, Trichiaceae and Arcyriaceae, whereas several families occur only sporadically (e.g. Acytosteliaceae, Echinosteliopsidaceae; small n) (Fig. 2).

### Taxa included

**Table:** 

Rank	Scientific Name	
phylum	Eumycetozoa	
class	Dictyosteliomycetes	
family	Dictyosteliaceae	
family	Acytosteliaceae	
class	Myxomycetes	
family	Stemonitidaceae	
family	Physaraceae	
family	Arcyriaceae	
family	Clastodermataceae	
family	Dianemataceae	
family	Trichiaceae	
family	Cribrariaceae	
family	Didymiaceae	
family	Stemonitaceae	
family	Dianemaceae	
family	Dictydiaethaliaceae	
family	Echinosteliaceae	
family	Elaeomyxaceae	
family	Tubiferaceae	
family	Hemitrichiaceae	
family	Liceaceae	
family	Listerellaceae	
family	Reticulariaceae	
class	Protosteliomycetes	
family	Ceratiomyxaceae	
family	Echinosteliopsidaceae	
family	Protosteliaceae	
family	Cavosteliidae	

## Traits coverage

**Figure 3.**
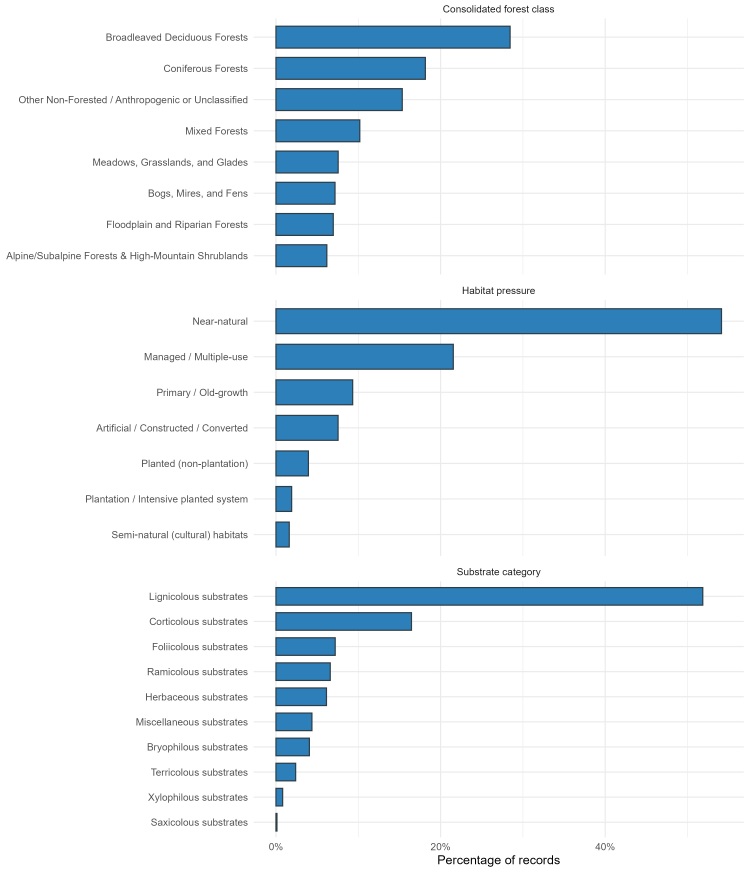
Trait coverage across consolidated forest classes, substrate categories and habitat‑pressure classes; bar lengths indicate the proportion of records mapped to each controlled label.

Trait information is recorded in Darwin Core topography fields and in *dynamicProperties* as minified JSON with explicit units in key names (Table 2). Elevation is held as *minimumElevationInMetres* and *maximumElevationInMetres* (metres), with the source string preserved separately for transparency. Environmental and forest context variables are standardised for cross‑study comparability and downstream modelling, with rounding rules enforced at ingestion (temperature to one decimal, pH to two decimals and precipitation/stand age as integers). Trait representation indicates broad coverage across consolidated forest classes (Table 3), substrates (Table 4) and management‑pressure categories (Fig. 3, Table 5). Observed ranges (dataset): *minimumElevationInMetres* −28–2750 m; *maximumElevationInMetres* 0–3000 m; *airTemperature_C* −3.3–21°C; *annualPrecipitation_mmYr* 132–2000 mm yr⁻¹; *pH* 3.35–10.00; *standAge_yr* discrete values 35–210 yr. pH values derive predominantly from moist‑chamber incubations; source decimal commas were harmonised to numeric dots with two‑decimal rounding for analysis.

**Figure 4.**
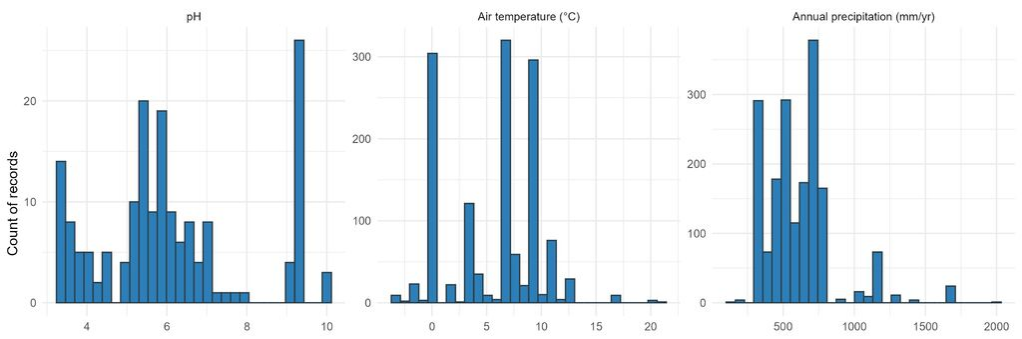
Environmental coverage for pH, air temperature (°C) and annual precipitation (mm yr⁻¹) shown as histograms (bins as in dataset workflow); medians are indicated to aid interpretation.

Continuous covariates span wide environmental space (Fig. 4): pH 3.35–10.00 (median ≈ 5.8; n = 173), air temperature −3.3–21°C (median ≈ 6.6°C; n = 1361) and annual precipitation 132–2000 mm yr⁻¹ (median ≈ 588 mm yr⁻¹; n = 1813).

## Temporal coverage

**Data range:** 1857-1-01 – 2025-8-01.

### Notes

**Figure 5.**
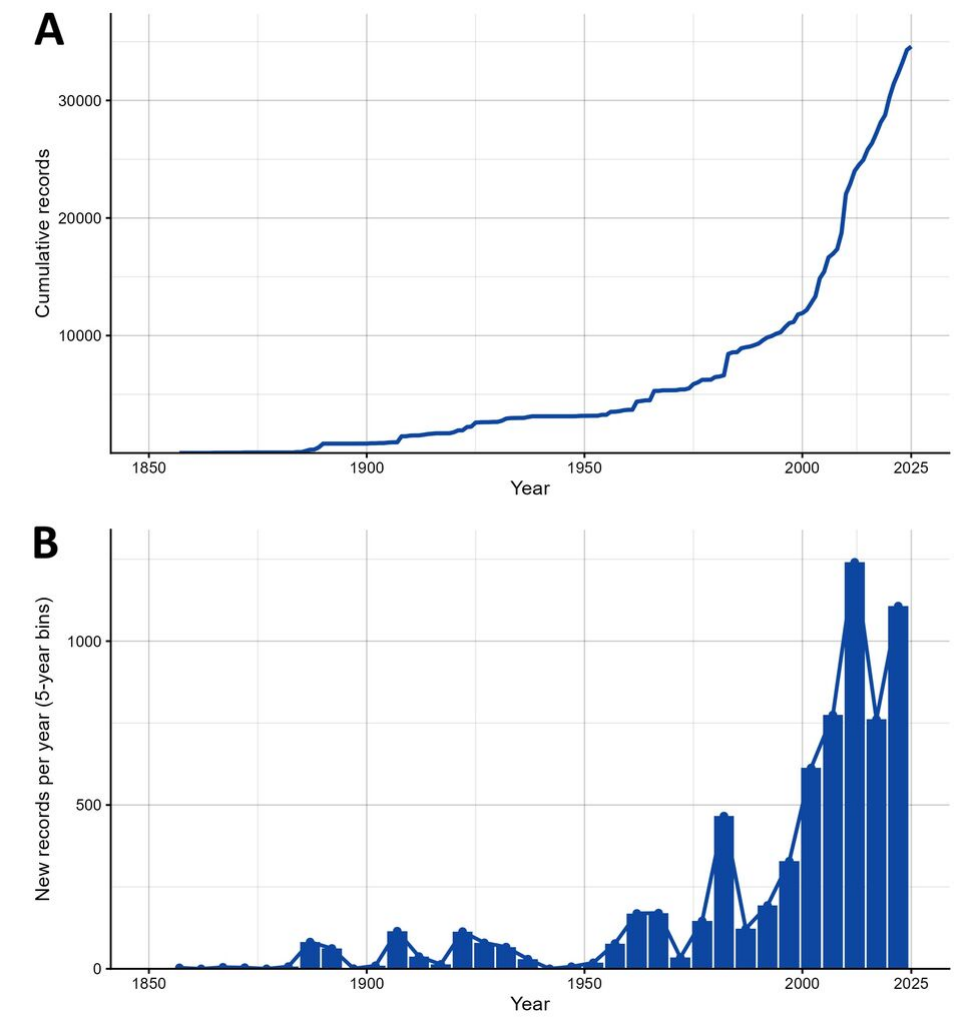
**(A)** Cumulative Eumycetozoa records by year (1857–2025), reflecting the historical growth of the literature-derived database; **(B)** Annual additions aggregated into five-year bins (dark-blue bars; values expressed as records per year), with a connecting line joining bin mid-points.

**Figure 6.**
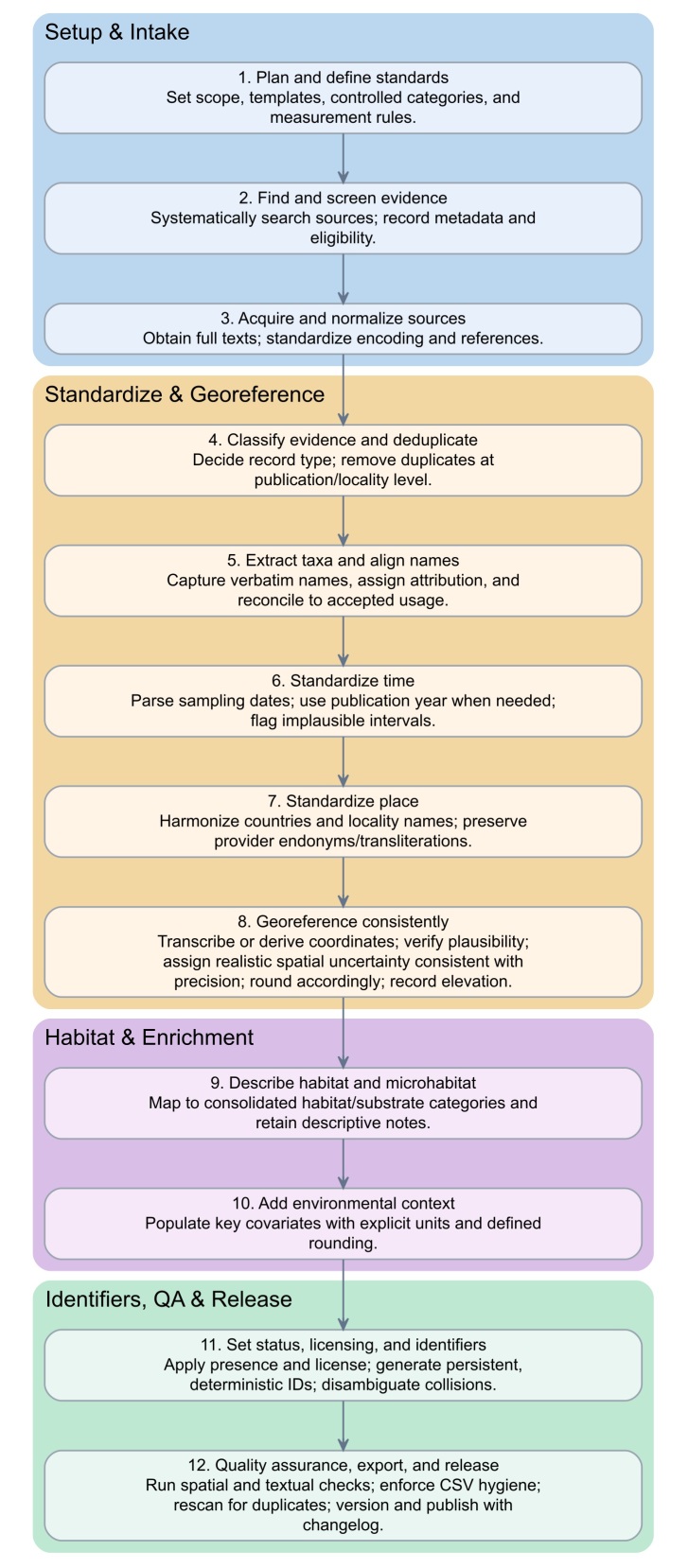
Schematic overview of the occurrence data curation workflow from source intake to versioned release.

Temporal coverage is represented by *eventDate*, *year*, *month* and *day*, spanning historical to present records with mixed precision, including ISO‑like single dates and bounded intervals. In the supplied material, *eventDate* ranges from 1857 to 2025-08-01; where only a publication year was available, that year served as a proxy with month/day left blank. The data mobilisation window was February 2022–August 2025; cleaning and harmonisation to GBIF/Darwin Core occurred from June to October 2025. Record accumulation accelerates markedly in recent decades (Fig. 5).

## Usage licence

### Usage licence

Other

### IP rights notes

All occurrence records are released under the Creative Commons Attribution 4.0 International licence, encoded in the licence field as the short form “CC‑BY‑4.0”. Full bibliographic provenance is supplied per record in *associatedReferences* to support proper attribution when reusing the dataset.

## Data resources

### Data package title

Georeferenced Checklist and Occurrence Dataset of Slime Moulds (Eumycetozoa) Across Central and Eastern Europe Emphasising Forest Ecosystems

### Resource link

https://doi.org/10.15468/jqukxm

### Alternative identifiers

https://ipt.pensoft.net/resource?r=eumycetozoa_central_eastern_europe

### Number of data sets

1

### Data set 1.

#### Data set name

Georeferenced Checklist and Occurrence Dataset of Slime Moulds (Eumycetozoa) Across Central and Eastern Europe Emphasising Forest Ecosystems

#### Data format

Comma‑separated values (CSV) implementing the Darwin Core Occurrence core; environmental covariates serialised as minified JSON in dynamicProperties.

#### Character set

UTF‑8 (Unicode); LF line endings; diacritics preserved; numeric fields use the dot as the decimal separator.

#### Download URL

https://ipt.pensoft.net/archive.do?r=eumycetozoa_central_eastern_europe&v=1.2

#### Description

This dataset assembles georeferenced, presence‑only occurrence records and a taxonomically standardised regional checklist of slime moulds (Eumycetozoa) from 16 countries across Central and Eastern Europe Pawłowicz 2025. Coverage emphasises forest ecosystems and complements each record with harmonised descriptors of substrate, stand type and management pressure. Environmental attributes (including elevation, precipitation, pH, stand age and consolidated forest class) enable comparative ecological and biogeographic analyses, forest‑focused modelling and gap mapping. Full source citations are provided for all records.

**Data set 1.** 

Column label	Column description
occurrenceID	Persistent unique identifier with a 36-character base UUID/GUID (UUID v5) generated deterministically from normalised bibliographic and locality tokens and stable under georeference updates.
basisOfRecord	Nominal evidence type with two case‑sensitive values —HumanObservation (literature‑derived field records without a cited voucher) and MaterialCitation (records citing examined material/vouchers).
occurrenceStatus	Presence state, uniformly “PRESENT”, confirming that all rows represent confirmed presences.
licence	Short‑form reuse licence code applied to every record: CC‑BY‑4.0.
associatedReferences	Harvard‑style reference string for the source (authors, year, title, outlet, pagination/identifier), optionally including DOI/URL.
eventDate	Sampling/observation date encoded as ISO‑like single dates.
year	Four‑digit calendar year parsed from eventDate.
month	Integer month (1–12) parsed from eventDate.
day	Integer day of month (1–31) parsed from eventDate.
country	Full English sovereign‑state name from the dataset’s 16‑country Central and Eastern Europe domain (Russian Federation records are European part only).
countryCode	ISO‑3166‑1 alpha‑2 uppercase country code corresponding to country.
stateProvince	Provider‑supplied subnational administrative unit (heterogeneous levels and endonyms retained; not harmonised).
locality	Harmonised Latin‑script site description using established English exonyms where available or consistent transliteration, constructed from verbatimLocality.
verbatimLocality	Exact locality string as printed in the source, preserving native language/script, diacritics and punctuation.
decimalLatitude	Geographic latitude in signed decimal degrees (dot separator), supplied only when localisable to ± 10 km and rounded to match coordinatePrecision.
decimalLongitude	Geographic longitude in signed decimal degrees (dot separator), supplied only when localisable to ± 10 km and rounded to match coordinatePrecision.
geodeticDatum	Geodetic reference datum for coordinates, uniformly “WGS84” in the delivered dataset.
coordinateUncertaintyInMetres	Non‑zero integer estimate of positional uncertainty (metres) accompanying any coordinates, documenting georeferencing precision.
coordinatePrecision	Plain‑decimal degree precision of the coordinates, taking one of six values (0.1, 0.01, 0.001, 0.0001, 0.00001, 0.000001) that reflect the reported resolution.
georeferencedBy	Personal name(s) of the individual(s) responsible for georeferencing or, where coordinates were copied verbatim, the publication authors.
georeferenceVerificationStatus	Binary georeferencing quality flag with values “verified” or “requires verification”.
minimumElevationInMetres	Minimum elevation in metres; single elevation statements are duplicated to this and maximumElevationInMetres, with the original string kept in verbatimElevation.
maximumElevationInMetres	Maximum elevation in metres paired with minimumElevationInMetres, derived from the same source statement where only one value is given.
scientificName	Accepted taxon name used for indexing, generally including authorship and year as curated against Eumycetozoa.com (GBIF backbone as fallback).
scientificNameAuthorship	Full nomenclatural authorship string associated with scientificName.
verbatimIdentification	Original taxonomic identification as printed in the source, retaining spelling, authorship format, qualifiers and infraspecific ranks.
nameAccordingTo	Authority to which the name usage is aligned, standardised to “Eumycetozoa.com” throughout the dataset.
identifiedBy	Name(s) of the identifier(s) of the taxon; where not stated in the source, the publication’s author(s) are recorded.
kingdom	Higher‑rank field uniformly set to “Protozoa”.
phylum	Higher‑rank field uniformly set to “Mycetozoa”.
class	Taxonomic class with three permissible values: “Dictyosteliomycetes”, “Myxomycetes” or “Protosteliomycetes”.
order	Taxonomic order assigned as one of eleven levels recognised in the dataset.
family	Taxonomic family assigned as one of 24 families.
genus	Generic epithet only (single token, with no authorship or rank qualifiers).
specificEpithet	Species‑level epithet in lower case, recorded without the generic name or authorship.
taxonRank	Rank at which the identification is asserted, restricted to “genus” or “species” in this dataset.
dynamicProperties	Minified JSON container for environmental and forest covariates (airTemperature_C, annualPrecipitation_mmYr, pH, standAge_yr, microhabitat, forestTypeDetailed, consolidatedForestCategory, substrateCategory, habitatPressure).

## Additional information

Conflicts of interest: none declared.

## Supplementary Material

2E187B77-01E7-5ED6-BA32-3F0A3A15F437 10.3897/BDJ.13.e175486.suppl1 Supplementary material 1 Dataset Bibligoraphy Data type Bibliography Brief description Dataset bibliography comprising of 528 bibliographic entries. File: oo_1460854.docx https://binary.pensoft.net/file/1460854 Tomasz Pawłowicz

## Figures and Tables

**Table 1.** Countries represented and ISO 3166 1 alpha 2 codes.

**Country**	**Code**	**Country**	**Code**
Austria	AT	Liechtenstein	LI
Belarus	BY	Lithuania	LT
Czechia	CZ	Moldova	MD
Estonia	EE	Poland	PL
Germany	DE	Russia (European part)	RU
Hungary	HU	Slovakia	SK
Latvia	LV	Slovenia	SI
Switzerland	CH	Ukraine	UA

**Table 2.** Environmental and forest centric attributes (storage, units and usage). Rounding policy at ingestion: temperature 0.1°C; pH 0.01; precipitation and stand age as integers.

**Field / key**	**Storage**	**Units / format**	**Normalisation & rounding**	**Analytical role**
*minimumElevationInMetres*	DwC topography	m (integer)	Single values duplicated to min/max when only one elevation reported	Elevational context for habitat comparisons
*maximumElevationInMetres*	DwC topography	m (integer)	As above	Elevational range per record
*airTemperature_C*	*dynamicProperties*	°C (numeric)	Rounded to 1 decimal at ingestion	Microclimatic covariate in forest settings
*annualPrecipitation_mmYr*	*dynamicProperties*	mm yr⁻¹ (integer)	Integer totals	Macro‑climatic covariate; stratifies moist vs. dry forests
*pH*	*dynamicProperties*	dimensionless	Decimal commas from sources normalised to numeric dots; rounded to two decimals	Substrate chemistry; particularly relevant for conifer‑derived detritus
*standAge_yr*	*dynamicProperties*	years (integer)	Integer; no fractions	Indicator of forest structural maturity
*microhabitat*	*dynamicProperties*	verbatim free text	Source phrasing retained (substrate, host taxon, decay state, height)	Fine‑scale context for substrate‑dependent taxa
*forestTypeDetailed*	*dynamicProperties*	verbatim free text	Syntaxa/management wording preserved	High‑resolution stand characterisation
*consolidatedForestCategory*	*dynamicProperties*	categorical (8 fixed labels)	Controlled vocabulary; one label per record	Broad class stratification across forests and allied habitats
*substrateCategory*	*dynamicProperties*	categorical (10 fixed labels)	Controlled vocabulary; one label per record	Substrate‑level contrasts
*habitatPressure*	*dynamicProperties*	categorical (7 fixed labels)	Controlled vocabulary; one label per record	Management/pressure gradien

**Table 3.** Consolidated forest classes (*consolidatedForestCategory*): authoritative category definitions and representative syntaxa, with Natura 2000 Annex I codes.

**Category**	**Detailed description of the category**	**Examples**
Broadleaved Deciduous Forests	Mesic to oligotrophic temperate broadleaved woodlands dominated by beech, oak, lime, hornbeam; exclude conifer‑dominated or even‑aged conifer plantings.	Tilio‑Carpinetum (typical/mesic/oligotrophic); Dentario glandulosae‑Fagetum; Galio odorati‑Fagetum; Luzulo pilosae‑Fagetum; Stellario‑Carpinetum; sessile‑oak forests (e.g. *Potentillo albae*‑*Quercetum petraeae*); Carici albae‑Fagetum; Natura 2000: 9110, 9130, 9150, 9160, 9170, 9180.
Coniferous Forests	Conifer‑dominated stands (natural or managed), including montane/subalpine belts and monocultures.	Pine forests (e.g. *Peucedano*‑*Pinetum*); montane/subalpine spruce; spruce monocultures; *Polysticho‑Piceetum* and related conifer communities. Natura 2000: 9410, 91T0, 91U0, 91R0.
Mixed Forests	Co‑dominated deciduous–conifer systems without a single dominant group.	Mixed pine forests (e.g. *Serratulo‑Pinetum*); beech–fir–spruce mixtures; *Polysticho*‑*Piceetum* with co‑dominant conifers and broadleaves (no single dedicated Annex I code; local overlaps with 9130, 9170, 9410, 91T0).
Alpine/Subalpine Forests & High‑Mountain Shrublands	High‑elevation belts at or above treeline, including krummholz and dwarf‑shrub systems.	*Pinus mugo* krummholz zone; snow‑bed shrub assemblages; ericaceous layers (e.g. *Vaccinium myrtillus*) in high mountains. Natura 2000: 4060, 4070, 95A0.
Bogs, Mires and Fens	Peat‑forming or waterlogged systems (bog woodlands, fen/carr), where mire conditions dominate hydrology and substrate.	*Vaccinio uliginosi*‑*Pinetum*; *Sphagno girgensohnii*‑*Piceetum*; peatland alder carrs (*Ribo nigri*‑*Alnetum*, *Sphagno squarrosi*‑*Alnetum*); bog birch (*Betula pubescens*) stands; Natura 2000: 91D0, 7110, 7120, 7140.
Floodplain and Riparian Forests	Riverine/alluvial systems shaped by periodic flooding and fluvial dynamics.	Alder–ash alluvial forests (e.g. *Circaeo*‑*Alnetum*); mineral alluvial alder woodlands; willow thickets within river corridors; floodplain terraces. Natura 2000: 91E0, 91F0.
Meadows, Grasslands and Glades	Non‑forest open habitats including semi‑natural hay meadows, grazed grasslands, subalpine meadows and intra‑forest glades.	Montane open meadows; mown/grazed semi‑natural meadows; subalpine meadows; intra‑forest clearings and glades. Natura 2000: 6510, 6520, 6230.
Other Non‑Forested / Anthropogenic or Unclassified	Sites lacking clear vegetation typology or heavily modified/urban settings; residual class when mapping is inconclusive.	Parks, lawns, road verges, abandoned industrial sites, waterlogged areas of uncertain vegetation, mixed urban greenspace (no dedicated Annex I code).

**Table 4.** Standardised substrate (microhabitat) categories (*substrateCategory*): operational definitions and typical tokens/examples.

**Category**	**Detailed description of the category**	**Examples**
Corticolous substrates	Bark of living or recently dead woody plants (outer periderm of trunks, stems, large branches).	“on bark”, “trunk surface”, “bark of *Fagus sylvatica*”.
Lignicolous substrates	Structural xylem in any decay stage (logs, stumps, standing trunks; deciduous or coniferous).	“on log”, “on decayed stump”, “coarse woody debris”.
Ramicolous substrates	Fine woody twigs/branches, attached or fallen, generally < 2 cm diameter.	“on small twig”, “branch litter”.
Foliicolous substrates	Foliar organs (leaves, needles) in canopy or as recent litter.	“on fallen leaves”, “on spruce needles”.
Bryophilous substrates	Living moss gametophytes and moss‑covered substrata.	“on moss cushions”, “mossy rock/wood”.
Herbaceous substrates	Aerial parts of non‑woody vascular plants (grasses, forbs, ferns, dwarf shrubs), alive or senescent.	“dead stems of grasses”, “on herb layer”.
Terricolous substrates	Upper soil horizons, humus layers and surface litter on the forest floor.	“on soil”, “forest floor litter”.
Saxicolous substrates	Exposed mineral substrata (rocks, stones, boulders), bare or cryptogam‑colonised.	“on rock face”, “on boulder”.
Xylophilous substrates	Anthropogenically processed wood (timber, boards, constructions) undergoing weathering/decay.	“on fence board”, “on wooden bridge”.
Miscellaneous/Unspecified	Rare, atypical or indeterminate substrata not assignable to the above categories.	“decaying organic matter (unspecified)”, “substrate not recorded”.

**Table 5.** Habitat-pressure (management intensity) classes (*habitatPressure*): assignment criteria and diagnostic field indicators with examples.

**Category**	**Detailed description of the category**	**Examples**
Primary / Old‑growth (Natural, lowest pressure)	No logging history; multi‑cohort structure; large deadwood volumes; undisturbed hydrology; no planting or regular spacing.	Indicators recorded in sources (e.g. “multi‑cohort, high deadwood; no planting; intact hydrology”).
Near‑natural (very low pressure)	Predominantly native composition/structure with minor legacy of low‑intensity use; occasional selective felling; deadwood present.	“selective felling traces”, “near‑natural stand with deadwood present”.
Semi‑natural (cultural) — low pressure	Extensive hay‑mowing or grazing; coppice legacies; open‑wooded meadows; low inputs; infrequent disturbance.	“hay meadow/grazing”, “coppice legacy”, “open‑wooded meadow”.
Managed / Multiple‑use (moderate pressure)	Active silviculture (thinning, rotations); mixed‑use forests; moderate stand homogenisation; altered yet functional processes.	“thinning/rotation regime”, “mixed‑use stand”, “moderate homogenisation”.
Planted (non‑plantation) — elevated pressure	> 50% planted/seeding origin, but lacking plantation regularity (heterogeneous spacing, mixed cohort ages).	“planted origin > 50%”, “heterogeneous spacing”, “mixed ages”.
Plantation / Intensive planted system — high pressure	Uniform, even‑aged stands; regular spacing; 1–2 species; short rotations; intensive interventions.	“regularly spaced monoculture”, “even‑aged cohort”, “short‑rotation stand”.
Artificial / Constructed / Converted — very high pressure	Parks, lawns, embankments, engineered substrates, urban greenspace; substantially modified soils/hydrology.	“urban park/lawn”, “embankment”, “engineered substrate”.
