# Supplementary material for: Georeferenced checklist and occurrence dataset of slime moulds (Eumycetozoa) across Central and Eastern Europe emphasising forest ecosystems
Source: Biodivers Data J. 2025 Nov 25;13:e175486. doi: 10.3897/BDJ.13.e175486 (PMC12673333; doi:10.3897/BDJ.13.e175486)
Supplement: Supplementary material 1 — Dataset Bibligoraphy [file bdj-13-e175486-s001.docx]

1. Adamonytė G. (2009). Gojaus miško gleivainiai. Lietuvos lokalinių tyrimų projektas Jieznas–Stakliškės (LLT)
2. Adamonytė, G.; Vimba, E., 2005. Notes on a collection of myxomycetes by K. Starcs in the herbarium of the University of Latvia. In: XVI Symposium of Mycologists and Lichenologists of the Baltic States, 21–25 September 2005, Cēsis, Latvia, pp. 43–47
3. Adamonytė, G. (1999) ‘Myxomycetes of the genus Diachea Fr. in Lithuania’, Botanica Lithuanica, 5(1), pp. 85–89
4. Adamonytė, G. (2000). New data on Estonian myxomycete biota. Folia Cryptogamica Estonica, 36, 7–9
5. Adamonytė, G. (2003). Myxomycetes of the genus Symphytocarpus Ing et Nann.-Bremek. in Lithuania. Botanica Lithuanica, 9(1), 55–63
6. Adamonytė, G. (2003). Trichia papillata, a new coprophilous myxomycete species. Mycotaxon, 87, 379–384
7. Adamonytė, G. (2006) New findings of myxomycetes in Latvia. Botanica Lithuanica, 12(1), 57–64
8. Adamonytė, G. (2006). The genus Echinostelium (Myxomycetes) in Lithuania. Acta Mycologica, 41(2), 169–176
9. Adamonytė, G. (2007). Myxomycetes of the genus Clastoderma in Lithuania. Botanica Lithuanica, 13(1), 27–32
10. Adamonytė, G. (2010) Lithuanian Stemonitales (Myxomycetes). Genera Amaurochaete and Brefeldia. Botanica Lithuanica, 16(2), 75–82
11. Adamonytė, G. (2013). Biržų girios gleivainiai
12. Adamonytė, G. (2020). A checklist of Latvian myxomycetes. Botanica, 26(2), 197–200
13. Adamonytė, G.; Iršėnaitė, R.; Motiejūnaitė, J.; Taraškevičius, R.; Matulevičiūtė, D. (2013). Myxomycetes in a forest affected by great cormorant colony: a case study in Western Lithuania. Fungal Diversity, 59(1), 131–146
14. Adamonyte, G.; Kastanje, V. (2011). Myxomycetes of the island of Saaremaa, Estonia. Folia Cryptog. Estonica, 48, 1–4
15. Adamonyte, G.; Mitchell, D. W. (2000). Notes on the Distribution of Licea clarkii B. Ing (Myxomycetes). Stapfia, 73, 77–80
16. Adamonytė, G.; Motiejūnaitė, J.; Iršėnaitė, R. (2016). Crown fire and surface fire: effects on myxomycetes inhabiting pine plantations. Science of The Total Environment, 572, 1431–1439
17. Adamonytė, G.; Vimba, E. (2004). Unpublished myxomycete collection of K. R. Kupffer. Folia Cryptogamica Estonica, 40, 1–6
18. Afanasyev, K.E.; Demakov, Y.P.; Bekmansurov, M.V.; Bogdanov, G.A.; Bogdanova, L.G.; Dyachkova, N.Yu.; Isaev, A.V.; Knyazev, M.N.; Kotlyakova, E.A.; Lavrova, O.V.; Oleneva, T.V.; Prokopyeva, L.V.; Ryzhkov, A.A.; Safin, M.G. (2009). Изучение естественного хода процессов, протекающих в природе, и выявление взаимосвязей между отдельными частями природного комплекса. Летопись природы за 2008 год. Государственный природный заповедник «Большая Кокшага», 15, 355 pp
19. Akulov, O. Yu.; Usichenko, A. S. (2020-08). Preliminary data about fungi and fungus-like organisms of the Dnistrovskyi Canyon National Nature Park. Chornomorski Botanical Journal, 16(2), 152–170
20. Amosov P.; Aleksandrova A.; Bucharitsin P.; Golovachev I.; Zemlyanskaya I.; Zmitrovitch I.; Kaganov V.; Karpenko N.; Kapralov S.; Kulakov V.; Kutlusurina G.; Morgun D.; Mukhanov A.; Novozhilov Yu.; Polynova G.; Popov A.; Popov E.; Rebriev Yu.; Safronova I.; Svetasheva T. (2012-01). Condition and long-term changes of natural environment in Bogdinsko-Baskunchaksky Reserve. Volgograd: IPK "Tsaritsyn"
21. Antonín, V. (1991). Zajímavé druhy naší mykoflóry. Mykologické listy, 42, 3–6
22. Antonínová, J.; Antonín, V.; Vlašín, M.; Vlašínová, H.; Suchý, M.; Máchalová, Z. (1985). IP CHÚ Bosonožský hájek. Manuscript, AOPK ČR
23. Apschner, C.; Duda, M.; Greilhuber, I.; Gross, H.; Hörweg, C.; Moog, O.; Reitmeier, W.; Scheiblhofer, J. (2023-03-22). Natur in Pressbaum: Ergebnisse zum Tag der Artenvielfalt 2019. Biosphärenpark Wienerwald Management
24. Barsukova, T.N. (2001-01). Myxomycetes of the Aliokhin Central Chernozem Biosphere Reserve. Mycology and Phytopathology, 35(1), 12–14
25. Barsukova, T.N.; Dunaev, E.A. (1997). An annotated list of slime moulds (Myxomycota) from Moscow Region. Mikologiya i fitopatologiya, 31(2), 1–8
26. Barsukova, T.N.; Prokhorov, V.P.; Gmoshinskii, V.I.; Chizhov, A.O. (2010-09-29). Myxomycetes in forest parks of Moscow, Moscow region, and some areas of the Kaluga region. Moscow University Biological Sciences Bulletin, 65(3), 116–118
27. Barsukova, T.N.; Vinogradskaya, E.N.; Akimova, M.F. (2006). Myxomycetes in forest parks in Moscow. Mikologiya i fitopatologiya, 40(3), 186–189
28. Baudyš, E.; Picbauer, R. (1923). Šestý příspěvek ku květeně moravských hub. Sborník Klubu přírodovědeckého v Brně, 5, 56–70
29. Baudyš, E.; Picbauer, R. (1924). Sedmý příspěvek ku květeně moravských a slezských hub. Sborník Klubu přírodovědeckého v Brně, 6(1), 71–89
30. Baudyš, E.; Picbauer, R. (1925). Příspěvek ke květeně hub Československé republiky. I. = Addenda ad floram Čechoslovakiae mycologicam I. Brno: Fytopathologická sekce (Publikace Fytopathologické sekce Zemského výzkumného ústavu zemědělského v Brně; 34), 25 pp
31. Bäumler, J. A. (1888). Fungi Schemnitzenses. Ein Beitrag zur ungarischen Pilzflora. Verhandlungen der Zoologisch-Botanischen Gesellschaft in Wien, 38, 707–720
32. Bäumler, J. A. (1890). Beiträge zur Cryptogamenflora des Pressburger Comitates. Pilze II. Verhandlungen des Vereines für Naturkunde zu Presburg, Neue Folge 7, 25–90
33. Bäumler, J. A. (1890). Fungi Schemnitzenses. Ein Beitrag zur ungarischen Pilzflora. II. Verhandlungen der Zoologisch-Botanischen Gesellschaft in Wien, 40, 139–148
34. Bäumler, J. A. (1903). Beiträge zur Cryptogamen-Flora des Presburger Comitates. Die Pilze. IV. Theil. Verhandlungen des Vereine für Naturkunde zu Presburg, 14, 31–88
35. Behrič, S. (2015) Raznolikost pravih sluzavk (Myxomycetes) v okolici Mengša. Diplomsko delo, Univerza v Ljubljani, Biotehniška fakulteta, Ljubljana
36. Bělohoubek, J. (ed.) (2001). Přírodovědný průzkum Údolí Hasiny u Lipence. ČSOP Hasina, Louny
37. Benike, L. A. (1915). Pervye svedeniya o flore slizistykh gribov Khar'kovskoy i Kurskoy guberniy. Protokoly Obshchestva Ispytateley Prirody pri Imperatorskom Khar'kovskom Universitete, Vypusk III (1914), III–V
38. Biosphärenpark Wienerwald Management (2015). Natur in Hernals – Ergebnisse zum Tag der Artenvielfalt 2014. Biosphärenpark Wienerwald Management GmbH, Tullnerbach
39. Biosphärenpark Wienerwald Management (2022). Natur in Währing – Ergebnisse zum Tag der Artenvielfalt 2020. Biosphärenpark Wienerwald Management GmbH
40. Blanár, D.; Mihál, I. (2002). Mykoflóra okolia Revúcej I (Slovenské rudohorie - Revúcka vrchovina) / Mycoflora in the Vicinity of Revúca Town I (The Slovenské Rudohorie - Revúcka Vrchovina Mountains)
41. Błoński, F. (1890). Wyniki poszukiwań florystycznych skrytokwiatowych dokonanych w ciągu lata r. 1889 w obrębie 5-ciu powiatów Królestwa Polskiego. Pamiętnik Fizyjograficzny, 10, 129–190
42. Błoński, F.; Drymmer, K. (1889). Sprawozdanie z wycieczki botanicznej, odbytej do Puszczy Białowieskiej, Ladzkiej i Świsłockiej w 1888 roku. Pamiętnik Fizyjograficzny, Botanika i Zoologija, 9, 55–115
43. Błoński, F.; Drymmer, K.; Ejsmond, A. (1888). Sprawozdanie z wycieczki botanicznej odbytej do Puszczy Białowieskiej w lecie 1887 roku. Pamiętnik Fizjograficzny, 8, 59–155
44. Bochynek, A. (2015). First Polish records of Myxomycetes rare in Europe. Acta Societatis Botanicorum Poloniae, 84(4), 443–448
45. Bochynek, A. and Drozdowicz, A., 2012. Biota śluzowców (Myxomycetes) lasu użytkowanego gospodarczo w okolicy przysiółka Wyrchczadeczka (Beskid Śląski). Sylwan, 156(1), pp. 57–63
46. Bochynek, A.; Drozdowicz, A. (2011). Dead wood as microhabitat for myxomycetes in selected forest communities in the Polish Carpathian Mts. Roczniki Bieszczadzkie, 19, 165–179
47. Bodonyi, N. and Tóth, S. (2004) Myxomycetes data from the Őrség National Park and environs of Budapest (Hungary). Mikológiai Közlemények, Clusiana, 43(1–3), 9–14
48. Bodyagin, V. V.; Barsukova, T. N. (2009). Myxomycetes isolated from aquatic habitats of Moscow city and Moscow Region. Mikologiya i fitopatologiya, 43(4), 281–283
49. Bolla, J. (1857). Die Pilze der Presburger Flora. Verhandlungen des Vereine für Naturkunde zu Presburg, 2(2), 43–71
50. Borg Dahl, M.; Shchepin, O.; Schunk, C.; Menzel, A.; Novozhilov, Y. K.; Schnittler, M. (2018-08-03). A four year survey reveals a coherent pattern between occurrence of fruit bodies and soil amoebae populations for nivicolous myxomycetes. Scientific Reports, 8, 11662
51. Borscow, E. (1869). Ein Beitrag zur Pilzflora der Provinz Cernigow. Bulletin de l’Académie Impériale des Sciences de St.-Pétersbourg, 13, 219–245
52. Borzov, N. I.; Bortnikov, F. M.; Matveev, A. V.; Gmoshinskiy, V. I. (2021). First data on plasmodial slime moulds (Myxomycetes = Myxogastrea) of Rdeysky Nature Reserve (Novgorod Region, Russia). Novosti sistematiki nizshikh rastenii, 55(2), 361–377
53. Borzov, N. I.; Gmoshinskiy, V. I. (2021). Results of the study of the myxomycete biota of the Polistovsky Nature Reserve. Proceedings of the Mordovia State Nature Reserve named after P. G. Smidovich, 28, 235–237
54. Borzov, N.I. & Gmoshinskiy, V.I., 2024. Rare species of Myxomycetes of Polistovo-Lovatskaya bog system (European part of Russia). Bulletin of the Moscow Society of Naturalists. Biological Series, 129(3), 43–56
55. Buch, K.; Schnittler, M.; Leontyev, D. (2023-10-02). A record of Ceratiomyxa hemisphaerica from Germany. Slime Molds, 4, V4A6
56. Burel, J.; Janda, V.; Landa, J. (2005). Zpráva o provedeném mykologickém průzkumu na územích NPR Karlštejn a NPR Koda v CHKO Český kras v období 1.1.2004 až 10.10.2005. Manuscript, AOPK ČR, Praha
57. Bykhalova, O. N. (ed.) (2017). Наземные и прилегающие морские экосистемы полуострова Абрау: структура, биоразнообразие и охрана. Научные труды, Том 4. Utrish State Nature Reserve
58. Čáp, J. (2004). Průzkum PR Pavlino údolí. Mykologický monitoring CHKO Labské pískovce. [Manuscript report]. Agentura ochrany přírody a krajiny ČR
59. Cejp, K. (1952). Další nálezy hlenky Lycogala flavo-fuscum (Ehrenb.) Rost. u nás. Česká Mykologie, 6(8-10), 156–159
60. Cejp, K. (1962) Beitrag zur Mykoflora der Schleimpilze (Myxomycetes), namentlich Westböhmens / Příspěvek k mykoflóře hlenek (Myxomycetes) Čech, zejména západních. Sborník Národního muzea v Praze, Řada B – Přírodní vědy (Acta Musei Nationalis Pragae, Series B – Historia Naturalis), 18(3), 61–80
61. Cejp, K. (1963-01-14). Podivná hlenka ve zplesnivělém herbáři. Česká Mykologie, 17(1), 47–48
62. Čelakovský, L. (1890). České Myxomycety. Arch. Přírod. Výzk. Čech, VII(5), 1–57
63. Chachuła, P., Melke, A., Ruta, R. & Szołtys, H. (2021). Myxomycete‑Coleoptera associations in the Polish Carpathians. Journal of Natural History, 55(27–28), 1749–1768
64. Clissmann, F.; Fiore-Donno, A. M.; Hoppe, B.; Krüger, D.; Kahl, T.; Unterseher, M.; Schnittler, M. (2015-05-07). First insight into dead wood protistan diversity: a molecular sampling of bright-spored Myxomycetes (Amoebozoa, slime-moulds) in decaying beech logs. FEMS Microbiology Ecology, 91(6)
65. Czernyadjeva, I. V.; Afonina, O. M.; Davydov, E. A.; Doroshina, G. Ya.; Dugarova, O. D.; Etylina, A. S.; Filippov, I. V.; Freydin, G. L.; Galanina, O. V.; Himelbrant, D. E.; et al. (2020-04). New cryptogamic records. 5. Novosti sistematiki nizshikh rastenii, 54(1), 261–286
66. Czernyadjeva, I. V.; Davydov, E. A.; Efimova, A. A.; Gogorev, R. M.; Himelbrant, D. E.; Kotkova, V. M.; Kuzmina, E. Yu.; Leostrin, A. V.; Moroz, E. L.; Neshataeva, V. Yu.; Notov, A. A.; Novozhilov, Yu. K.; Paukov, A. G.; Popova, N. N.; Potemkin, A. D.; Stepanchikova, I. S.; Storozhenko, Yu. V.; Yakovchenko, L. S.; Yurchak, M. I.; Volosnova, L. F.; Zhurbenko, M. P.; Zyatnina, M. V. (2021). New cryptogamic records. 7. Novosti sistematiki nizshikh rastenii, 55(1), 249–277
67. Czernyadjeva, I. V.; Kotkova, V. M.; Zemlyanskaya, I. V.; Novozhilov, Yu. K.; Vlasenko, A. V.; Vlasenko, V. A.; Blagoveshchenskaya, E. Yu.; Georgieva, M. L.; Notov, A. A.; Himelbrant, D. E.; Muchnik, E. E.; Urbanavichene, I. N.; Aristarkhova, E. A.; Bocharnikov, M. V.; Ismailov, A. B. (2018). New cryptogamic records. 2. Novosti Sistematiki Nizshikh Rastenii, 52(1), 209–223
68. Dämon, W. (2001). Notizen zur Pilzflora des Bundeslandes Salzburg (1). Linzer biologische Beiträge, 33(2), 723–796
69. Dämon, W.; Klenke, F.; Krisai-Greilhuber, I. (2013). Fundliste der 37. Internationalen Mykologischen Dreiländertagung in Tamsweg 2013. Österreichische Zeitschrift für Pilzkunde, 22, 121–162
70. Döbbeler, P.; Remler, P. (1976). Über einige neue oder bemerkenswerte Myxomyceten der Steiermark. Mitteilungen des Naturwissenschaftlichen Vereines für Steiermark, 106, 131–141
71. Dredor, D.; Szmatona-Túri, T. (2023). New data to Hungarian slime molds (Protozoa: Myxomycetes). Natura Somogyiensis, 41, 57-66
72. Dredor, D.; Szmatona-Túri, T. (2023). NEW RECORDS OF SLIME MOLD (PROTOZOA: MYXOMYCETES) SPECIES FOR HUNGARIAN FLORA AND DATA OF ITS SUBSTRATES. Acta Biologica Plantarum Agriensis, 11(2), 26
73. Drozdowicz, A. (1997). Studies on Myxomycetes in the Pieniny National Park I. New species for the PNP. Acta Mycologica, 32(2), 287–291
74. Drozdowicz, A. (2005). Materials to the chorology of Myxomycetes in the Bieszczady National Park. Roczniki Bieszczadzkie, 13, 261–276
75. Drozdowicz, A., Szolc, P., Bochynek, A. and Salamaga, A. (2012) Myxomycetes of the Lipówka reserve in the Niepołomice Old Growth Forest (S Poland). Acta Mycologica, 47(1), pp. 97–107
76. Drozdowicz, A.; Paczyńska, G.; Pięta, M. (2007). Changes in the biota of slime moulds (Myxomycetes) in the Korytania gorge in Ojcow National Park. Prądnik. Prace i Materiały Muzeum Im. Prof. Władysława Szafera, 17, 61–70
77. Drozdowski, I. (2013). Natur in Altenmarkt an der Triesting – Ergebnisse zum Tag der Artenvielfalt 2011. Biosphärenpark Wienerwald Management GmbH
78. Društvo študentov biologije (2011-07). Raziskovalni tabor študentov biologije Slovenske gorice – Sveti Jurij ob Ščavnici 2011
79. Dudka, I. A.; Kuzub, V. V.; Romanenko, E. A. (1999). Myxomycetes of the Yalta Mountain-Forest Nature Reserve (Crimea, Ukraine). Mikologiya i Fitopatologiya, 33(5), 307–313
80. Dudka, I. O.; Heluta, V. P.; Andrianova, T. V.; Hayova, V. P.; Tykhonenko, Yu. Ya.; Prydiuk, M. P.; Holubtsova, Yu. I.; Krivomaz, T. I.; Dzhagan, V. V.; Leontyev, D. V.; Akulov, O. Yu.; Syvokon, O. V. (2009). Fungi of the nature reserves and national nature parks of Left-Bank Ukraine. M.G. Kholodny Institute of Botany, NAS of Ukraine. ISBN 978-966-02-4996-7
81. Dudka, I. O.; Kryvomaz, T. I. (1996). New species of myxomycetes from the Ukrainian Carpathians. Ukrainian Botanical Journal, 53(6), 710–716
82. Dudka, I. O.; Kryvomaz, T. I. (2013). Myxomycetes in the virgin beech and old-aged spruce forests of National Nature Park "Bewitched Land" (Ukrainian Carpathians). Studia Biologica, 7(2), 107–118
83. Dudka, I. O.; Leontyev, D. V. (2011). Myxomycetes in virgin forests of Carpathian Biosphere Reserve. Studia Biologica, 5(1), 45–56
84. Dudka, I. O.; Romanenko, K. O. (2006). Co-existence and interaction between myxomycetes and other organisms in shared niches. Acta Mycologica, 41(1), 99–112
85. Dudka, I.; Kryvomaz, T. (2008). Myxomycetes of the Ichnyanskyi National Natural Park of Ukraine. Mycology and Phytopathology, 42(5), 432-439
86. Dudka, I.O.; Kryvomaz, T.I. (2005). Myxomycetes of the Desnyansk-Starogut National Nature Park. Scientific Bulletin of Chernivtsi National University. Biology, 260, 111–117
87. Dudka, I.O.; Kryvomaz, T.I. (2010). Myxomycetes in ecotopes and plant communities of Dunais’ky biosphere reserve. Chornomors’k Botanical Journal, 6(1), 54–66
88. Dudka, I.O.; Kryvomaz, T.I. (2011). Summer aspect of myxomycete biota in the national nature park 'Prypiat'-Stokhid'. Chornomorski Botanical Journal, 7(1), 67–83
89. Dvořáková, R. (1999). Hlenky (Myxomycetes) vypěstované ve vlhkých komůrkách. In Jankovský, L.; Krejčík, R.; Antonín, V. (eds.), Houby a les, 157–160. Brno
90. Dvořáková, R. (2002) ‘Myxomycetes in Bohemian Karst and Hřebeny Mts.’, Czech Mycology, 53(4), pp. 319–349
91. Egri, K. (2008). Újabb adatok ritka nagygombafajok előfordulásáról Zemplénben. Folia historico-naturalia Musei Matraensis, 32, 19–25
92. Eiser, R. and Follmann, G. (1984) Zur Kryptogamenflora und Kryptogamenvegetation des Naturschutzgebietes Urwald Sababurg im Reinhardswald (Nordhessen). I. Die Schleimpilze (Myxomycophyta). Hessische Floristische Briefe, 33, pp. 51–58
93. Eiser, R.; Flatau, L.; Schirmer, P. (1980). Myxomyceten aus Nordhessen. Zeitschrift für Mykologie, 46(1), 15–18
94. Engel, H. and Hechler, J. (1983) Nahezu 100 Schleimpilzarten in Nordwestoberfranken. Die Pilzflora Nordwestoberfrankens, 7, 26–33
95. Erastova, D. A. (2015). Nival myxomycetes (Myxomycetes) of Northwestern Russia and the Northwestern Caucasus (Нивальные миксомицеты (Myxomycetes) Северо-Запада России и Северо-Западного Кавказа). Candidate of Biological Sciences thesis, Komarov Botanical Institute (BIN RAS), St. Petersburg, Russia
96. Erastova, D.A. & Novozhilov, Y.K. (2015) Nivicolous myxomycetes of the lowland landscapes of the Northwest of Russia. Mycology and Phytopathology, 49(1), 9–18
97. Erastova, D.A.; Novozhilov, Y.K.; Schnittler, M. (2017). Nivicolous myxomycetes of the Khibiny Mountains, Kola Peninsula, Russia. Nova Hedwigia, 104(1-3), 85–110
98. Evstigneev, O. I.; Fedotov, Yu. P.; Sitnikova, E. F. (2007). Tsarstvo griby: nastoyashchie griby, slizeviki, lishayniki zapovednika 'Bryanskiy les'. Bryansk, 56 pp
99. Fefelov, K. A. (2006). An annotated check-list of the myxomycetes in the Visimskii Biosphere Reserve ( Аннотированный список миксомицетов Висимского государственного заповедника). Ecological investigations in the Visimskii Biosphere Reserve, 336–339
100. Fefelov, K. A. (2010). Basic check-list of the Myxomycetes from Uzhno-Uralsky State Reserve. Trudy Tigirekskogo zapovednika, 3: Mountain ecosystems of South Siberia: study, conservation and rational nature use. Barnaul
101. Fefelov, K. A. (2010). Myxomycetes of the Urals. Mikologiya i Fitopatologiya, 44(4), 340–351
102. Feng, Y.; Schnittler, M. (2015-08-11). Sex or no sex? Group I introns and independent marker genes reveal the existence of three sexual but reproductively isolated biospecies in Trichia varia (Myxomycetes). Organisms Diversity & Evolution, 15(4), 631–650
103. Feng, Y.; Schnittler, M. (2017). Molecular or morphological species? Myxomycete diversity in a deciduous forest in northeastern Germany. Nova Hedwigia, 104(1-3), 359–380
104. Fiore-Donno, A. M.; Novozhilov, Y. K.; Meyer, M.; Schnittler, M. (2011-08-01). Genetic Structure of Two Protist Species (Myxogastria, Amoebozoa) Suggests Asexual Reproduction in Sexual Amoebae. PLoS ONE, 6(8), e22872
105. Fischerová, M. (2021). Hlenky (Myxomycetes) na území PP Bobří soutěska [Bachelor’s thesis]. Univerzita J. E. Purkyně v Ústí nad Labem, Přírodovědecká fakulta
106. Fischerová, M. (2023). Hlenky (Myxomycetes) v okolí obce Janovice u Kravař. Diploma thesis, Jan Evangelista Purkyně University in Ústí nad Labem, Faculty of Science
107. Flatau, L. (1982). Myxomyceten aus Nord-Hessen – I. Ein neuer Myxomycet aus dem Reinhardswald bei Kassel. Zeitschrift für Mykologie, 48(2), 257–259
108. Flatau, L. (2000). Die Gattung Licea im Fuldatal bei Kassel (Myxomyceten). Stapfia, 73, 63–74
109. Flatau, L. and Schirmer, P. (1994) ‘Neue Myxomyceten-Funde in Nordhessen und Deutschland’, Zeitschrift für Mykologie, 60(1), pp. 253–274
110. Flatau, L.; Massner, W.; Schirmer, P. (1987). Myxomyceten aus Nordhessen – IV. Ein neuer Myxomycet aus der Umgebung von Kassel. Zeitschrift für Mykologie, 53(1), 145–149
111. Flatau, L.; Schirmer, P. (1983). Myxomycetes from Northern Hessen - II. A new Myxomycete from the Surroundings of Kassel. Zeitschrift für Mykologie, 49(2), 179–182
112. Flatau, L.; Schirmer, P. (2004). Neue Myxomyceten aus Deutschland. Zeitschrift für Mykologie, 70(2), 187–206
113. Flugrová, P. (2022). Hlenky (Myxomycetes) na území Kostelního lesa města Varnsdorf [Bachelor's thesis]. Univerzita J. E. Purkyně v Ústí nad Labem, Přírodovědecká fakulta
114. Frélich, Z.; Lazebníček, J. (2010). Mykologický inventarizační průzkum v PR Karlovice sever
115. Frišová, L. (2019) Hlenky (Myxomycetes) v údolí potoka Rytina u Sebuzína. Bakalářská práce. Ústí nad Labem: Univerzita Jana Evangelisty Purkyně v Ústí nad Labem, Přírodovědecká fakulta
116. Geltman, D. V.; Himelbrant, D. E.; Konechnaya, G. Yu.; Kotkova, V. M.; Luknitskaya, A. F.; Potemkin, A. D.; Safronova, T. V.; Smirnova, S. V.; Stepanchikova, I. S.; Andreev, M. P.; Belyakova, R. N.; Boldina, O. N.; Gagarina, L. V.; Glazkova, E. A.; Gogorev, R. M.; Doronina, A. Yu.; Doroshina, G. Ya.; Efimov, P. G.; Zhakova, L. V.; Katayeva, O. A.; Kovalchuk, N. A.; Kuznetsova, E. S.; Mikhaylova, T. A.; Morozova, O. V.; Novozhilov, Yu. K.; Popov, E. S.; Sorokina, I. A.; Spirin, V. A. (2018). Vascular plants, bryophytes, algae, lichens, fungi and slime molds needed in regional conservation measures in the Leningrad Region. Botanicheskii Zhurnal, 103(6), 764–811
117. Gmoschinskiy, V. I.; Bogatova, P. D.; Matveev, A. V. (2018). The results of the study of species diversity myxomycetes, Poria Guba 2017. Letopis' prirody Kandalakshskogo zapovednika za 2017 god (annual report), book 63, vol. 1, pp. 61–65
118. Gmoshinskiy, V. I. (2014). An annotated checklist of the myxomycetes of the Poriya Guba bay (White Sea): primary report. Chronicle of Nature of the Kandalaksha Nature Reserve for 2013 (annual report), 2, 9–31
119. Gmoshinskiy, V. I. (2017). Results of studying myxomycetes diversity on the territory of 'Utrish' Reserve in 2016. In: Kalinina, S. Yu.; Bykhalova, O. N. (eds), Terrestrial and adjacent marine ecosystems of the Abrau peninsula: structure, biodiversity and protection. Proceedings, 4, 134–140. I.V. Kazenin, Moscow
120. Gmoshinskiy, V. I.; Bortnikov, F. M.; Matveev, A. V.; Novozhilov, Y. K. (2020). New data on Myxomycetes of Lazovsky State Nature Reserve (Far East, Russia). Botanica Pacifica: a journal of plant science and conservation, 9(1), 155–164
121. Gmoshinskiy, V. I.; Kireeva, N. I. (2023). First data on nivicolous myxomycetes in the “Bitsevsky forest” natural and historical park (Moscow, Russia). Mikologiya i fitopatologiya, 57(5), 372–377
122. Gmoshinskiy, V. I.; Kireeva, N. I. (2024). Red list of Tver region. Third edition. Chapter 1. Myxomycetes. Red Data Book of Tver Region, 3rd ed. Strategy ECO; Tver Print Yard
123. Gmoshinskiy, V. I.; Kireeva, N. I. (2024). Studies of the nivicolous myxomycetes in lowland habitats
124. Gmoshinskiy, V. I.; Kireeva, N. I.; Sakulin, S. V.; Matveev, A. V.; Novozhilov, Y. K. (2025-06-30). Critical revision of the myxomycete (Myxomycetes, Myxomycota) collection at the Mycological herbarium LEP. I. Yaroslavl Region. Turczaninowia, 28(2), 36–50
125. Gmoshinskiy, V. I.; Matveev, A. V. (2016). Season dynamics of sporulation of myxomycetes in Moscow city and Moscow Region. Mikologiya i Fitopatologiya, 50(3), 139–147
126. Gmoshinskiy, V. I.; Matveev, A. V. (2021). Kadastrovye svedeniya o Tsentral'no-Lesnom gosudarstvennom prirodnom biosfernom zapovednike za 2017–2020 gg. Central Forest State Nature Biosphere Reserve, Zapovedny
127. Gmoshinskiy, V. I.; Matveev, A. V.; Lazareva, O. A. (2021). New Data on Myxomycetes (=Myxogastrea, Mycetozoa) from Dagestan State Nature Reserve (North Caucasus, Russia). Bulletin of Moscow Society of Naturalists. Biological series, 126(4), 36–49
128. Gmoshinskiy, V. I.; Mishulin, A. A. (2022). New and Rare Species of Myxomycetes of the Genus Stemonaria (Myxomycetes = Myxogastrea) for Russia. Bulletin of the Moscow Society of Naturalists. Biological Series, 127(1), 11–18
129. Gmoshinskiy, V. I.; Mishulin, A. A.; Matveev, A. V. (2019). First record of Didymium projectile (Myxomycetes) in Russia. Bulletin of the Moscow Society of Naturalists. Biological Series, 124(4), 34–36
130. Gmoshinskiy, V. I.; Peters, D. A.; Kireeva, N. I.; Perevedentseva, L. G. (2025). First records of Badhamia ovispora (Physarales, Myxomycetes) in Russia. Novosti sistematiki nizshikh rastenii, 59(1), F1–F9
131. Gmoshinskiy, V.I. and Kireeva, N.I. (2022) New data on myxomycetes of the Prioksko-Terrasny Nature Reserve (Moscow Region). In: Scientific research and ecological monitoring on specially protected natural areas of Russia and adjacent countries: conference proceedings of the Central Forest State Nature Biosphere Reserve. Moscow: KMK Scientific Press, pp. 240–245
132. Gmoshinskiy, V.I. and Matveev, A.V. (2019) First data on Myxomycetes of Polistovsky Nature Reserve (Pskov Region). Novosti sistematiki nizshikh rastenii, 53(2), pp. 279–290
133. Gmoshinskiy, V.I., Bukhtoyarova, N.Yu. & Matveev, A.V., 2017. Изучение видового разнообразия миксомицетов южного лесничества Центрально‑Лесного государственного природного биосферного заповедника. Летопись природы Центрально‑Лесного государственного природного биосферного заповедника, 56, 192–225
134. Gmoshinskiy, V.I., Matveev, A.V., Gubanov, E.S., Bortnikov, F.M. and Dunayev, E.A. (2020) ‘Critical revision of the Myxomycetes collection of Young Naturalists Club of Zoological Museum of Moscow State University’, Botanica Pacifica, 9(2), pp. 175–190
135. Gönczöl, J.; Révay, Á. (1981). Data to the knowledge of microscopic fungi of the Ócsa Nature Conservation Area. Studia Botanica Hungarica, 15, 19–24
136. Gorunova, A.V. & Ebel, M.A., 2018. Представители рода Stemonitis в биоте миксомицетов природного парка «Волго‑Ахтубинская пойма». В: Рогова, Н.В. (ред.), День фармацевтического факультета ВолгГМУ – 2017: сборник материалов. Волгоград: Изд‑во ВолгГМУ, 76–80. ISBN 978‑5‑9652‑0505‑9
137. Gottsberger, G. (1966). Die Myxomyceten der Steiermark mit Beiträgen zu ihrer Biologie. Nova Hedwigia, 12(1/2), 203–296
138. Gutwiński, R. (1901). Materiały do flory śluzowców (Myxomycetes) Galicji. Sprawozdanie Komisyi Fizyograficznej, 35, 73–75
139. Hardtke, H.-J., Siegel, M. and Wähner, H. (1991) Zur Pilzflora des Elbhügellandes und der angrenzenden Gebiete (4. Beitrag: Myxomyceten). Boletus, 15(1), 25–32
140. Hausknecht, A., Jaklitsch, W.M. & Krisai-Greilhuber, I. (2003) Rezente Pilzfunde aus Osttirol. Österreichische Zeitschrift für Pilzkunde, 12, 153–192
141. Hazslinszky, F. (1884). Előmunkálatok Magyarhon gombavirányához. Mathematikai és Természettudományi Közlemények, 19, 63–118
142. Hechler, J. (1991). Pilzneufunde in Nordwestoberfranken 1990, III. Teil. Myxomyceten (Schleimpilze). Die Pilzflora Nordwestoberfrankens, 14–15, 51–53
143. Hechler, J.; Engel, H. (1989). Pilzneufunde in Nordwestoberfranken 1988, III. Teil. Myxomyceten (Schleimpilze). Die Pilzflora Nordwestoberfrankens, 13, 27–30
144. Hemerka, A. (1996). Mykologická inventarizace NPP Šejval 1995–1996. Inventarizační průzkum. AOPK ČR, Pardubice
145. Hennings, P. (1903). Beitrag zur Pilzflora des Gouvernements Moskau. Hedwigia, Beiblatt 42, 108–118
146. Hennings, P. (1906). Dritter Beitrag zur Pilzflora des Gouvernements Moskau. Hedwigia, 45, 22–33
147. Heyden, K. K. (1899). Zur Pilzflora des Gouvernements Moskau. Hedwigia, 38, 269–273
148. HLISNIKOVSKÝ D. (2020) Terénní zápisky - náhodná pozorování
149. Hollós, L. (1913) Kecskemét vidékének gombái. Mathematikai és Természettudományi Közlemények, 32(3), 149–325
150. Hollós, L. (1933). Szekszárd vidékének gombái (Fungi regionis Szekszárdiensis). Matematikai és Természettudományi Közlemények, 37(2), 1–205
151. Hoppe, T. (2013-07). Molecular diversity of myxomycetes near Siegen (Germany). Mycoscience, 54(4), 309–313
152. Hoppe, T., Ammon, L. and Moll, J.K. (2014) ‘Diversity and sporocarp development of lignicolous myxomycetes in young timber forests of western Germany’, Österreichische Zeitschrift für Pilzkunde, 23, pp. 131–141
153. Hoppe, T.; Müller, H.; Kutschera, U. (2010). A new species of Physarum (Myxomycetes) from a boreal pine forest in Thuringia (Germany). Mycotaxon, 114(1), 7–14
154. Horsák, M., Kočí, K., Kočí, M., Kroča, J., Kuras, T., Kubešová, S., Vašutová, M. and Wolfová, J. (2003) Komplexní botanicko‑zoologická inventarizace NPR Razula v CHKO Beskydy
155. Hullen, M.; Kirzinger, A. (2005). Artenbericht 2005 (mit Listen u. a. 'Mycetes – Pilze'). Nationalpark Harz, 110 pp
156. Huňáček, M. (2022). Hlenky (Myxomycetes) v horní části Bezručova údolí u Chomutova. Diplomová práce, Univerzita Jana Evangelisty Purkyně v Ústí nad Labem, Přírodovědecká fakulta
157. Ing, B. (1990). New records of myxomycetes in Estonia. Proceedings of the Estonian Academy of Sciences, Biology, 39(4), 271–276
158. Ing, B. (2024). The myxomycetes of Switzerland and Liechtenstein: A check-list and census catalogue of the recorded species. Miscellanea Mycologica, 1, Article 2
159. Ing, B., 1999. Corticolous Myxomycetes from Switzerland. Mycologia Helvetica, 10(2), pp.25–40
160. Inoue, M.; Woyzichovski, J.; López-Villalba, Á.; Shchepin, O.; Klahr, A.; Novozhilov, Y. K.; Schnittler, M. (2024-07-16). Using barcoding to reveal ecological patterns of nivicolous myxomycetes in the German Alps: How do they deal with varying snow conditions?. Fungal Ecology, 71, 101374
161. Iršėnaitė, R.; Adamonytė, G.; Daniele, I.; Kasparavičius, J.; Kutorga, E.; Stončius, D. (2013). Macromycetes and myxomycetes of Asveja Regional Park (Lithuania). Botanica Lithuanica, 19(1), 8–21
162. Jančovičová, S.; Godovičová, K.; Trojanovičová, L.; Vašková, Z.; Michalová, M.; Dušička, J.; Hrabovský, M.; Miškovic, J.; Mišíková, K. (2019). Let’s go to the field. Botanical excursion 3. Acta Botanica Universitatis Comenianae, 54, 39–56
163. Janik, P.; Ronikier, A. (2016). Meriderma species (Myxomycetes) from the Polish Carpathians: a taxonomic revision using SEM-visualized spore ornamentation. Acta Societatis Botanicorum Poloniae, 85(1), 3492
164. Jarocki, J. (1924) Śluzowce Puszczy Białowieskiej. Część I. Śluzowce z Rezerwatu Północnego [The Myxomycetes of the Great Białowieża-Forest. Part I. Slime-moulds from the Northern Protected Territory]. Acta Societatis Botanicorum Poloniae, 2(3), pp. 183–199
165. Jarocki, J. (1931). Mycetozoa from the Czarnohora mountains in the Polish Eastern Carpathians. Bull. Acad. Polon. Sci. Lett., Ser. B, 447–464
166. Jurc, D.; Ogris, N.; Piltaver, A.; Dolenc, A. (2004). Seznam vrst in razširjenost makromicet v Sloveniji z analizo stopnje ogroženosti. Gozdarski inštitut Slovenije, Ljubljana, 408 pp
167. Kadlec P. (2016). Myxomycota v okolí Kamenického Šenova
168. Kalinowska‑Kucharska, E. (1975) Materials for the flora of myxomycetes of central Poland. Acta Mycologica, 11(2), 93–99
169. Kireeva, N. I.; Botyakov, V. N.; Gmoshinskiy, V. I.; Novozhilov, Y. K. (2025). New species of the genus Physarum (Myxomycetes = Myxogastrea) for Russia. Bulletin of the Moscow Society of Naturalists. Biological Series, 130(1), 12–22
170. Klán, J. (1982). Mykoflóra SPR Jiřina. Inventarizační průzkum. AOPK ČR – Ústřední seznam ochrany přírody
171. Kloubec, B. (1990). Lesnický inventarizační průzkum navrhované SPR "Široké blato". Inventarizační průzkum
172. Kluša, J. (2023). Complete checklist of myxomycetes in Latvia. Acta Biol. Univ. Daugavp., 23(2), 127–151
173. Kochergina, A. V. (2020). First findings of myxomycetes on the territory of the Seym Regional Landscape Park. Biodiversity, Ecology and Experimental Biology, 22(2), 30–36
174. Kochergina, A.; Schnittler, M. (2025). Epiphytic and fimicolous myxomycetes on the island Hiddensee (Germany): rare species, new genotypes and unexpected ecological preferences. European Journal of Protistology, 100, 126153
175. Kochergina, A.V. & Markina, T.Y. (2021). Ecological assemblages of corticulous myxomycetes in forest communities of the North‑East Ukraine. Biosystems Diversity, 29(2), 94–101
176. Kochergina, A.V.; Leontyev, D.V. (2019-12-20). Addition to the species composition of myxomycetes of Shatskyi National Nature Park. Chornomorski Botanical Journal, 15(4), 371–381
177. Kociánová, M. (2019). Nivikolní hlenky
178. Kocourková, J. (1999) Lichenicolous fungi of the Czech Republic (The first commented checklist). Fossil Imprint / Acta Musei Nationalis Pragae, Series B – Historia Naturalis, 55(3–4), 59–169
179. Kofler, A. and Nowotny, W. (1992) Über einheimische Schleimpilze (Myxomycètes). Carinthia II, 182/102, 175–185
180. Kotelenets, N. N.; Barsukova, T. N. (2003). Myxomycetes and myxomycetophilous beetles in Oksky State Biosphere Reserve. Mycology and Phytopathology, 37(1)
181. Kotkova V.M.; Czernyadjeva I.V.; Davydov E.A.; Doroshina G.Ya.; Efimov D.Yu.; Efimova L.A.; Frolov I.V.; Gabiger Ya.I.; Glushchenko M.Yu.; Gorbunova I.A.; Himelbrant D.E.; Ignatenko M.E.; Kalinina L.B.; Kurbatova L.E.; Kushnevskaya H.V.; Lashchinsky N.N.; Lotiev K.Yu.; Moroz E.L.; Notov A.A.; Novozhilov Yu.K.; Otmakhov Yu.S.; Plikina N.V.; Popova N.N.; Potemkin A.D.; Putilina V.A.; Ryzhkova P.Yu.; Sambyla Ch.N.; Smirnova E.V.; Stepanchikova I.S.; Storozhenko Yu.V.; Troeva E.I.; Tsurykau A.G.; Vishnyakov V.S.; Vlasenko A.V.; Vlasenko V.A.; Volkova E.A.; Volosnova L.F.; Yakovchenko L.S.; Yatsenko-Stepanova T.N.; Zhuykov K.A.; Zueva A.S (2023-04-02). New cryptogamic records. 11. Novosti sistematiki nizshikh rastenii, 57(1), 155–204
182. Kotkova, V. M.; Beljakova, R. N.; Gorin, K. K.; Himelbrant, D. E.; Khanov, Z. M.; Kolganikhina, G. B.; Moroz, E. L.; Notov, A. A.; Novozhilov, Yu. K.; Popova, N. N.; Stepanchikova, I. S.; Zhdanov, I. S.; Zholobova, Zh. O. (2022). New cryptogamic records. 9. Novosti sistematiki nizshikh rastenii, 56(1), 203–220
183. Kotkova, V. M.; Morozova, O. V.; Novozhilov, Yu. K.; Popov, E. S.; Zhurbenko, M. P.; Zmitrovich, I. V. (2007). Preliminary list of Fungi and Myxomycetes of Leningrad region. St. Petersburg: Komarov Botanical Institute (TREEART LLC), 52 pp
184. Kotkova, V.M.; Afonina, O.M.; Belyakov, E.A.; Bobokalonov, K.A.; Bolsun, I.M.; Davydov, E.A.; Doroshina, G.Ya.; Dyachenko, A.P.; Erokhina, O.V.; Filippova, N.V.; Genkal, S.I.; Golubkov, V.V.; Gorbunova, I.A.; Goskova, S.M.; Himelbrant, D.E.; Ivchenko, T.G.; Kapitonov, V.I.; Khetagurov, Kh.M.; Kipriyanova, L.M.; Makryi, T.V.; Maksimov, A.I.; Mongush, Ch.B.; Moroz, E.L.; Moseev, D.S.; Nikolayev, I.A.; Norkulov, M.M.; Notov, A.A.; Plikina, N.V.; Popova, N.N.; Romanov, R.E.; Safronova, T.V.; Shadrina, S.N.; Smirnova, L.Ya.; Smirnova, M.A.; Stepanchikova, I.S.; Storozhenko, Yu.V.; Tsurykau, A.G.; Vaishlya, O.B.; Vilk, E.F.; Vishnyakov, V.S.; Vlasenko, A.V.; Vlasenko, V.A.; Vorzheva, V.V.; Yakovchenko, L.S. (2025-03-30). New cryptogamic records. 15. Novosti sistematiki nizshikh rastenii, 59(1), R1–R26
185. Kramoliš, J.; Tejklová, T. (2011). Výsledky inventarizačního průzkumu makromycetů Přírodní památka Kusá hora. Inventarizační průzkum
186. Krieglsteiner, L. (1995). Ergänzungen zur Myxomyceten-Fauna Deutschlands 1: Über die corticicolen Arten Badhamia versicolor A. Lister und Diderma chondrioderma (de Bary & Rost.) G. Lister. Rheinland-Pfälzisches PilzJournal, 5, 122–133
187. Krieglsteiner, L. (2000) ‘Nachträge zur “Myxomyceten-Fauna” Ostbayerns’, Mycologia Bavarica, 4, pp. 55–69
188. Krieglsteiner, L. (2000). Nivicole Myxomyceten im Hoch-Sauerland (Nordrhein-Westfalen, Deutschland) - Lamproderma pulveratum Bozonnet, Meyer & Poulain neu für Deutschland. Stapfia, 73, 131–133
189. Krieglsteiner, L. (2002) Pilze im NSG Sippenauer Moor bei Saal a. d. Donau (südwestlich Regensburg) – Resultate einer einjährigen Untersuchung. Regensburger Mykologische Schriften, 10, 67–133
190. Krisai-Greilhuber, I.; Koller, G. (2014). Pilze im Gesäuse – Der GEO-Tag 2013 im Haindlkar. Schriften des Nationalparks Gesäuse, 11, 86–98
191. Kruglikov, S. A. (2007). Slizeviki (Miksomitsety) zapovednika "Bryansky les". Tsarstvo griby: nastoyashchie griby, slizeviki, lishayniki zapovednika "Bryanskiy les". Bryansk
192. Krupa, J. (1886). Zapiski mykologiczne przeważnie z okolic Lwowa i z Tatr. Kosmos, 11, 370–399
193. Krupa, J. (1887). Zapiski mykologiczne z okolic Lwowa i Podtatrza. Sprawozdanie Komisji Fizjograficznej, 22(2), 12–100
194. Krupa, J. (1889). Zapiski mycologiczne z okolic Lwowa i z Karpat stryjskich. Sprawozdanie Komisyi Fizyograficznej, 23(1), 141–169
195. Kryvomaz T. I. (2009). Myxomycetes of the Cheremske Nature Reserve. Zapovidna sprava v Ukraini
196. Kryvomaz, T. and Dudka, I. (2014) ‘Рідкісні для України види міксоміцетів з гербарію Львівського національного університету імені Івана Франка’, Вісник Львівського університету. Серія біологічна, 65, pp. 97–106
197. Kryvomaz, T. I. (2004). Myxomycetes of the Shatsky National Park. Ukrainian Botanical Journal, 61(5), 45–53
198. Kryvomaz, T. I. (2004). Перші відомості про міксоміцети Черемського природного заповідника (Волинська область)
199. Kryvomaz, Tetyana (2014-02-28). First steps in myxomycete conservation activities. Fungal Conservation, Issue 4, 35–39
200. Krzemieniewska, H. (1937). Sluzowce zebrane w starym ogrodzie botanicznym we Lwowie. Kosmos, 62, 17–26
201. Krzysztofiak, L.; Krzysztofiak, A.; Romański, M. (2010). Świat śluzowców, grzybów i mszaków Wigierskiego Parku Narodowego. Stowarzyszenie "Człowiek i Przyroda"
202. Kuhnt, A. (2005). Observations on four rare species of Hemitrichia (Myxomycetes, Trichiales). Zeitschrift für Mykologie, 71(2), 165–178
203. Kuhnt, A. (2006). Nivicole Myxomyceten aus Deutschland (unter besonderer Berücksichtigung der bayerischen Alpen). Teil II. Zeitschrift für Mykologie, 72(2), 101–113
204. Kuhnt, A. (2007). Nivicole Myxomyceten aus Deutschland (unter besonderer Berücksichtigung der bayerischen Alpen). Teil I. Mycologia Bavarica, 9, 57–68
205. Kuhnt, A. (2010). Nivicole Myxomyceten aus Deutschland (unter besonderer Berücksichtigung der bayerischen Alpen). Teil V. Mycologia Bavarica, 11, 49–64
206. Kuhnt, A. (2011). Lamproderma lycopodiicola und L. nordica (Myxomycetes, Stemonitales), zwei neue nivicole Arten. Zeitschrift für Mykologie, 77(1), 71–88
207. Kuhnt, A. (2012). Myxomyceten im Naturwaldreservat "Schönwald" (Deutschland, Oberbayern). Zeitschrift für Mykologie, 78(1), 65–96
208. Kuhnt, A. (2014). Bemerkenswerte "Schleimpilze" (Amoebozoa, Myxomycetes) aus Deutschland: Neu- und Wiederfunde seltener Arten. Berichte der Bayerischen Botanischen Gesellschaft zur Erforschung der Flora, 84, 39–64
209. Kuhnt, A. (2019). Bemerkenswerte Myxomycetenfunde: Neue Arten, Neukombinationen und Nachweise seltener Arten – Teil 2. Berichte der Bayerischen Botanischen Gesellschaft zur Erforschung der Flora, 89, 139–222
210. Kuhnt, A., 2009. Nivicole Myxomyceten aus Deutschland (unter besonderer Berücksichtigung der bayerischen Alpen). Teil IV. Zeitschrift für Mykologie, 75(2), pp.189–230
211. Kuhnt, A.; Baumann, K.; Nowotny, W. (2014). Didymium tussilaginis (Berk. & Broome) Massee, Didymium vernum spec. nov., and Diacheopsis spec. – three hitherto overlooked foliicolous myxomycete species on Common Butterbur (Petasites hybridus). Zeitschrift für Mykologie, 80(1), 137–167
212. Kutorga, E., Adamonytė, G., Iršėnaitė, R., Kasparavičius, J., Markovskaja, S., Motiejūnaitė, J. & Treigienė, A. (2012) A checklist of mycobiota recorded in burnt and unburnt Pinus mugo plantations in the Curonian Spit (Lithuania). Botanica Lithuanica, 18(1), 66–79
213. Lásková, A. (2009). Inventarizační průzkum připravovaného ZCHÚ Čedičový vrch zaměřený na hlenky. Ms. Depon. in: knihovna Správy CHKO Labské pískovce, Děčín
214. Lásková, A. (2010) Hlenky – inventarizační průzkum NP České Švýcarsko na lokalitách Český vrch, Růžovský vrch, Divoká soutěska a Hauschengrund – Zlé díry. Závěrečná zpráva za rok 2010. Krásná Lípa: Správa NP České Švýcarsko (manuscript)
215. Lásková, A. (2011). Hlenky – inventarizační průzkum NP České Švýcarsko na lokalitě Růžovský vrch. Závěrečná zpráva za rok 2011. Manuscript, Správa NP České Švýcarsko, Krásná Lípa
216. Lavitska, Z. G. (1949). Materials for the flora of slime molds (Myxomycetes) of the Middle Dnieper region. Protocols of Kaniv biogeographic reserve, 7, 47-49
217. Lazebníček, J.; Frélich, Z. (2011). Vytvoření komplexního monitorovacího systému přírodního prostředí Moravskoslezského kraje – 2.17 Mykologický inventarizační průzkum v PR Suchá Dora, Závěrečná zpráva. Moravskoslezský kraj. 27 s
218. Lebedev, A. N. (2007). Некоторые итоги изучения флоры миксомицетов Тверской области. Вестник ТвГУ. Серия: Биология и экология, (5), 138–140
219. Lebedev, A. N.; Gmoshinskiy, V. I.; Buchtoyarova, N. Yu. (2017). New data on myxomycetes diversity in Central Forest Nature Reserve (Nelidovo area, Tver Region). Vestnik Tver State University. Series: Biology and Ecology, (1), 217–236
220. Lebedev, A. N.; Gmoshinsky, V. I. (2012). Distribution of Lycogala conicum Pers. (Myxomycetes) in Russia. Yaroslavl Pedagogical Bulletin, 3, 115–117
221. Lebedev, A. N.; Notov, A. A.; Korobkov, A. G. (2008). Myxomycetes of the Udomel’skij district of the Tver region. Vestnik TvGU. Series Biology and Ecology, 8, 136–142
222. Lebedev, A.N.; Notov, A.A. (2009). An annotated checklist of the slime moulds from the Botanical Garden of Tver State University. Tver State University Bulletin. Series: Biology and Ecology, 13, 186–192
223. Leontyev, D. V. (2006). New records of Myxomycetes in Ukraine (Myxomycota). Mycology and phytopathology, 40(3), 218–230
224. Leontyev, D. V. (2010). Plant community preferences of some myxomycete species in Gomolsha forests (Ukraine). Nauka i Studia (Poland), 4(28), 14–24
225. Leontyev, D. V. (2022). New data on the distribution of the rare myxomycete Tubifera dudkae (Reticulariaceae) in the context of the contribution of citizen science to the biodiversity monitoring. Chornomors'k. bot. z., 18(1), 71–78
226. Leontyev, D. V.; Akulov, O. Yu.; Dudka, I. O.; Kochergnia, A. V. (2019-01-18). FIRST RECORDS OF MYXOMYCETES IN THE HUTSULSHCHINA NATIONAL NATURE PARK (UKRAINIAN CARPATHIANS). Biologia ta valeologia, 20, 102–110
227. Leontyev, D. V.; Dudka, I. O.; Kocherhina, A. V.; Kryvomaz, T. I. (2010). Myxomycota of the National Nature Park "Synevyr". Ukrainian Botanical Journal, 67(4), 615–622
228. Leontyev, D. V.; Dudka, I. O.; Kryvomaz, T. I. (2009). Myxomycetes of the Podilski Tovtry National Park. Ukrainian Botanical Journal, 66(2), 240–249
229. Leontyev, D. V.; Dudka, I. O.; Malaniuk, V. B.; Kochergina, A. V. (2011). Myxomycetes of Halytskyi National Nature Park. Ukrainian Botanical Journal, 68(4), 604–617
230. Leontyev, D. V.; Dudka, I. O.; Malanyuk, V. B.; Van Hoof, J. P. M. (2013). Myxomycetes of the Gorgany Nature Reserve. Ukrainian Botanical Journal, 70(1), 94–102
231. Leontyev, D. V.; Eliasson, U.; Kochergina, A. V.; Morozova, I. I. (2009-12-28). New and rare myxomycetes of Ukraine. 1. East Forest-Steppe. Karstenia, 49(2), 61–67
232. Leontyev, D. V.; Kochergina, A. V. (2018). Myxomycetes of the Carpathian biosphere reserve in the collection of Professor I.O. Dudka, kept in the herbarium of H.S. Skovoroda Karkiv National Pedagogical University. Chornomorski Botanical Journal, 15(1), 80–85
233. Leontyev, D. V.; McHugh, R.; Fefelov, K. A.; Kochergina, A. V. (2011). New and rare Myxomycetes of Ukraine. 2. Southwest Crimea. Nova Hedwigia, 92(1-2), 245-256
234. Leontyev, D. V.; Moreno, G. (2011). Reticularia dudkae. A new myxomycete species from oak forests of eastern Ukraine. Boletín de la Sociedad Micológica de Madrid, 35, 85–94
235. Leontyev, D.V. (2006). Species composition of Myxomycetes (Myxomycota) in the Gomol’shanskie Lesa National Park (Ukraine). Mikologiya i Fitopatologiya, 40(2), 101–107
236. Leontyev, D.V. (2010) ‘Myxomycetes from the genera Stemonitis, Stemonitopsis and Stemonaria in Ukraine: identification and distribution’, Mycology and Phytopathology (Mikologiya i Fitopatologiya), 44(5), pp. 398–409
237. Leontyev, D.V. (2013). Myxomycetes from the genera Comatricha, Macbrideola and Paradiacheopsis in Ukraine: identification and distribution. Mikologiya i Fitopatologiya, 47(3), 159–168
238. Leontyev, D.V., Dudka, I.O., Kochergina, A.V. & Kryvomaz, T.I. (2012). New and rare Myxomycetes of Ukraine 3. Forest and forest‑steppe zone. Nova Hedwigia, 94(3–4), 335–354
239. Leontyev, D.V.; Yatsiuk, I.I.; Kochergina, A.V. (2020-06-30). Inclusion of myxomycetes in the Red Data Book of Ukraine: feasibility, selection criteria and recommended species. Ukrainian Botanical Journal, 77(3), 189–203
240. Lepšová, A. (2004). Mykologický průzkum NPR Chlumská stráň. Inventarizační průzkum. Agentura ochrany přírody a krajiny ČR, Praha
241. Lešák, L. (1994) Inventarizační průzkum a návrh opatření plánu péče pro návrh ZCHÚ Libějovický park. Manuscript (Ms.). Ústřední seznam ochrany přírody (ÚSOP)
242. Lotz-Winter, H.; Hofmann, T.; Kirschner, R.; Kursawe, M.; Trampe, T.; Piepenbring, M. (2011). Pilze im Botanischen Garten der Universität Frankfurt am Main. Zeitschrift für Mykologie, 77(1), 89–122
243. Luptakova, A. D. (2018). Миксомицеты Самарской области. In: Lomonosov-2018, International scientific conference of students and young scientists. Moscow State University
244. Ławrynowicz, M., Ślusarczyk, D. and Salamaga, A. (2011) ‘Revised data on the occurrence of myxomycetes in Central Poland’, Acta Mycologica, 46(2), pp. 223–232
245. Magnus, P. (1905). Die Pilze (Fungi) von Tirol, Vorarlberg und Liechtenstein. I. Myxomycetes
246. Magyar, D. & Tóth, S. (2003). Data to the Knowledge of the Microscopic Fungi in the Forests around Budakeszi (Buda Hills, Hungary). Acta Phytopathologica et Entomologica Hungarica, 38(1–2), 61–72
247. Manic, Ş. (2014). Contributions to taxonomic diversity research of macromycobiota of Moldova. Revista Botanică, 2(9), 52–63
248. Manic, Ș. (2015) Macromicetele din Republica Moldova (taxonomie, bioecologie, corologie). Doctor habilitat thesis, Academia de Științe a Moldovei, Chișinău
249. Matveev, A. V.; Gmoshinskiy, V. I. (2022). Myxomycetes (Myxomycetes) of the greenhouses of the Botanical Garden of Moscow State University 'Aptekarskiy Ogorod'. Green Journal – Bulletin of the Botanical Garden of Tver State University, 10, 3–11
250. Matveev, A. V.; Gmoshinskiy, V. I.; Prokhorov, V. P.; Kazantseva, E. S. (2018). The myxomycetes from botanical gardens of Moscow: N. V. Tsitsin botanical garden of the Russian Academy of Sciences and the Botanical garden of Moscow State University. Mikologiya i fitopatologiya (Mycology and Phytopathology), 52(2), 104–111
251. Matveev, A. V.; Gmoshinsky, V. I. (2017). ПЕРВЫЕ ДАННЫЕ О МИКСОМИЦЕТАХ ДАГЕСТАНА. Труды государственного природного заповедника «Дагестанский», 13, 20–27
252. Matveev, A. V.; Gmoshinsky, V. I.; Botyakov, V. N.; Novozhilov, Y. K. (2018). First records of Physarella oblonga (Myxomycetes) in Russia. Bulletin of the Moscow Society of Naturalists. Biological Series, 123(4), 66–77
253. Matveev, A. V.; Lebedev, A. N.; Gmoshinskiy, V. I. (2018). Results of long-term research of myxomycetes biota in the Tver State University Botanical Garden. Mikologiya i fitopatologiya, 52(2), 112–119
254. Melkumov, G. M. (2015). Видовой состав плазмодиальных миксомицетов (Myxomycetes) Новоусманского района Воронежской области
255. Meylan, C. (1908) ‘Contribution à la connaissance des myxomycètes du Jura’, Bulletin de la Société Vaudoise des Sciences Naturelles, 44(164), pp. 285–303
256. Meylan, C. (1910) ‘Myxomycètes du Jura [suite]’, Bulletin de la Société Vaudoise des Sciences Naturelles, 46(168), pp. 49–59
257. Meylan, C. (1914). Remarques sur quelques espèces nivales de myxomycètes. Bulletin de la Société Vaudoise des Sciences Naturelles, 50(182), 1–14
258. Meylan, C. (1920). Contribution à la connaissance des myxomycètes de la Suisse. Bulletin de la Société Vaudoise des Sciences Naturelles, 53(199), 451
259. Meylan, C. (1924). Recherches sur les Myxomycètes du Jura en 1921-22-23. Bulletin de la Société Vaudoise des Sciences Naturelles, 55(214), 237–244
260. Meylan, C. (1925). Note sur divers Myxomycètes du Jura et des Alpes. Bulletin de la Société Vaudoise des Sciences Naturelles, 56(216), 65–74
261. Meylan, C. (1926). Recherches sur les myxomycètes du Jura en 1925–26. Bulletin de la Société Vaudoise des Sciences Naturelles, 56(219), 319–328
262. Meylan, C. (1929). Recherches sur les myxomycètes en 1927-28. Bulletin de la Société Vaudoise des Sciences Naturelles, 57(223), 39–47
263. Meylan, C. (1931). Contribution à la connaissance des Myxomycètes du Jura et des Alpes. Bulletin de la Société vaudoise des sciences naturelles, 57(227), 297–300
264. Meylan, C. (1931-12-02). Les espèces nivales du genre Lamproderma. Bulletin de la Société Vaudoise des Sciences Naturelles, 57(228), 359–373
265. Meylan, C. (1933). Recherches sur les Myxomycètes du Jura 1930-31-32. Bulletin de la Société Vaudoise des Sciences Naturelles, 58(233), 81–90
266. Meylan, C. (1935). Recherches sur les Myxomycètes suisses en 1933–34. Bulletin de la Société Vaudoise des Sciences Naturelles, 58(236), 319–320
267. Meylan, C. (1937). Nouvelle contribution à la connaissance des myxomycètes du Jura et des Alpes. Bulletin de la Société Vaudoise des Sciences Naturelles, 59(244), 479–486
268. Meylan, Ch. (1916). Nouvelles contributions à l'étude des Myxomycètes du Jura. Bulletin de la Société Vaudoise des Sciences Naturelles, 51(191), 259–269
269. Mihál, I.; Blanár, D. (2007). Mykoflóra v oblasti magnezitového závodu Slovmag, Lubeník (Slovenské rudohorie – Revúcka vrchovina). Reussia, 4(1–2), 35–59
270. Mihál, I.; Blanár, D. (2014). Fungi and slime molds of alder and willow alluvial forests of the upper part of the Muránka river (central Slovakia). Folia oecologica, 41(2), 153–172
271. Mihál, I.; Blanár, D. (2016). Slizovky a huby (Myxomycota, Ascomycota, Basidiomycota) prírodnej rezervácie Fabova hoľa v Národnom parku Muránska planina. Natura Carpatica, LVII, 7–24
272. Mihál, I.; Blanár, D.; Glejdura, S. (2012). New, rare and less known slime molds and fungi (Myxomycota, Zygomycota, Ascomycota, Basidiomycota) found in Central Slovakia. Folia Oecologica, 39(2), 121–129
273. Mihál, I.; Blanár, D.; Glejdura, S. (2015). Enhancing knowledge of mycoflora (Myxomycota, Zygomycota, Ascomycota, Basidiomycota) in oak-hornbeam forests in the vicinity of the magnesite plants at Lubeník and Jelšava (central Slovakia). Thaiszia – J. Bot., 25(2), 121–142
274. Minter, D. W. (1981). Microfungi on needles, twigs and cones of pines in Czechoslovakia. Česká Mykologie, 35(2), 90–101
275. Miśkiewicz, A. (2001) Slime moulds occurring in the Bukowiec reserve (W Carpathians). Acta Mycologica, 36(1), 21–29
276. Miśkiewicz, A.; Drozdowicz, A. (1999). The new site of Diderma deplanatum and Diderma chondrioderma in the Pogórze Wiśnickie Region (S Poland). Acta Mycologica, 34(2), 299–304
277. Moesz, G. (1925). Fungi Hungariae I. Myxomycetes (Magyarország gombaflórája. I. Nyálkagombák). Folia Cryptogamica, 1(3), 111–200
278. Moesz, G. (1934). Gombák Magyarország északi részéből
279. Moesz, G. (1941). Dunántúli gombák (Pilze aus dem westlichen Gebiete Ungarns). A Magyar Biológiai Kutatóintézet Munkái, 13, 175–186
280. Moreno, G., López-Villalba, A., Castillo, A., Romanenko, K.O. and Leontyev, D.V. (2017) ‘Notes on some myxomycetes from Crimea (Ukraine)’, Mycotaxon, 132(3), pp. 649–663
281. Moreno, G.; Sánchez, A.; Meyer, M.; López-Villalba, Á.; Castillo, A. (2018). Revision of the nivicolous species of the genus Lepidoderma. Boletín de la Sociedad Micológica de Madrid, 42, 39–77
282. Moroz E.L.; Moroz A.E.; Moroz L.E. (2024). New records of myxomycetes in the reserve 'Yukhnovsky' (Belarus)
283. Moroz, E. L. (1996). Miksomitsety Belorusskogo Poozerya [Myxomycetes of the Belarusian Lakeland]. In Sokhranenie biologicheskogo raznoobraziya Belorusskogo Poozerya: Tezisy dokladov regional'noi nauchno-prakticheskoi konferentsii (Vitebsk), 145–146
284. Moroz, E. L. (2018). Myxomycetes of the spruce forests of the Narochansky National Park. In Problems of nature conservation organization of landscapes: Proceedings of the international scientific-practical conference (pp. 114–118). Novocherkassk
285. Moroz, E. L. (2023-10-10). Первое сообщение о миксомицетах (Myxomycetes) национального парка «Припятский» (Республика Беларусь). В: Мониторинг и оценка состояния растительного мира: материалы VI Международной научной конференции, Минск–Лясковичи, 10–13 октября 2023 г., p. 304
286. Moroz, E. L.; Novozhilov, Ju. K. (2024). Biota of myxomycetes in forests of National Park Narochansky. Botany (Research), Institute of Experimental Botany of the National Academy of Sciences of Belarus. Botany (research), 53
287. Moroz, E. L.; Novozhilov, Y. K. (1994). New and rare Myxomycetes species from Belarus. Mikologiya i fitopatologiya, 28(3), 21–27
288. Moroz, E. L.; Novozhilov, Y. K. (2010). First report on myxomycetes (Myxomycetes) of the National Park Belovezhskaya Pushcha (Belarus)
289. Moroz, E. L.; Novozhilov, Y. K. (2019). New and rare slime-molds (Myxomycetes) of the National Park “Narochansky” (Belarus). Novosti sistematiki nizshikh rastenii, 53(2), 307–314
290. Moroz, E. L.; Novozhilov, Y. K. (2021). Myxomycetes (Myxomycetes = Myxogastrea) of leaf litter of black alder forests of the Narachanski National Park (Belarus). Mycology and Phytopathology, 55(6), 423–430
291. Moroz, E. L.; Novozhilov, Y. K. (2021). Таксономическая структура биоты миксомицетов Национального парка «Нарочанский» (Республика Беларусь). Микология и фитопатология, 55(6), 457–466
292. Moroz, E. L.; Novozhilov, Yu. K. (2019-09-24). The first information about the myxomycetes of the Berezinsky Biosphere Reserve. In: Flora and vegetation in a changing world: problems of study, conservation and rational use (Proceedings of the International Scientific Conference, Minsk–Domzheritsy, 24–27 September 2019), pp. 116–119. Minsk: Kolorgrad. ISBN 978-985-596-426-2
293. Moroz, E.L. (2020). Licea pusilla Schrad. — новый для Беларуси вид миксомицетов (Myxomycetes). Minsk: Kolorgrad
294. Moroz, E.L. (2021) Pervye svedeniya o miksomycetakh landsaftnogo zakaznika “Vydritsa” (Respublika Belarus’) [First information about myxomycetes of the landscape reserve “Vydritsa” (Republic of Belarus)]
295. Moroz, E.L. and Novozhilov, Y.K. (2023) O myksomycetakh (klass Myxomycetes) natsional’nogo parka ‘Belovezhskaya Pushcha’ (Respublika Belarus’). In: Voronin, L.V., Kurakova, A.V., Shiryaeva, A.G. and Volobueva, S.V. (eds) Ekologiya gribov i gribopodobnykh organizmov: fakty, gipotezy, tendentsii: tezisy dokladov Vserossiiskoi nauchnoi konferentsii s mezhdunarodnym uchastiem, Yaroslavl, 12–14 October 2023. Yaroslavl: RIO YaGPU, p. 41
296. Moroz, E.L.; Moroz, A.E. (2021). FIRST INFORMATION ABOUT MYXOMYCETES OF THE BIOLOGICAL RESERVE "BOLMYANSKY" (Belarus)
297. Moroz, E.L.; Novozhilov, Yu.K. (2020). New records of myxomycetes for the Belarus. Novosti sistematiki nizshikh rastenii, 54(1)
298. Morozova, I. and Leontyev, D. (2010) Myxomycetes of Medobory Nature reserve (Ternopil region). Zapovidna sprava v Ukraini, 16(2), 40–44
299. Motiejūnaitė, J.; Buožytė, R.; Adamonytė, G.; Iršėnaitė, R.; Kasparavičius, J.; Kutorga, E.; Markovskaja, S.; Stakėnas, V.; Klyukina, E. (2018-05-18). Residual Effect of Induced Water Stress and Nitrogen Addition on the Mycobiota in Scots Pine Stands. Russian Journal of Ecology, 49(3), 226–231
300. Mrkos, O. (1927). První příspěvek k mykofloře Moravy. Sborník Klubu přírodovědeckého v Brně, 9, 69–78
301. Müller, H. (2005). Bemerkenswerte Myxomycetenfunde in Thüringen. Zeitschrift für Mykologie, 71(2), 211–220
302. Müller, H. (2007). Myxomycetes on Calluna vulgaris. Zeitschrift für Mykologie, 73(2), 245–250
303. Müller, H. (2008). Licea sinuatopicta sp. nov. und weitere Funde von corticolen Licea-Arten (Myxomycetes) in Thüringen. Zeitschrift für Mykologie, 74(2), 295–302
304. Müller, H. and Schulz, W. (2010) Myxomyceten an Fruchtständen von Alnus in Thüringen. Zeitschrift für Mykologie, 76(1), pp. 75–82
305. Müller, H., 2002. Beitrag zur Kenntnis und Verbreitung nivicoler Myxomyceten im Thüringer Wald. Zeitschrift für Mykologie, 68(2), pp.199–208
306. Müller, H.; Riemay, K.-H. (2010). Rote Liste der Schleimpilze (Myxomycetes) Thüringens (Naturschutzreport 26). Naturschutzreport, 26, 485–490
307. Müller, H.; Schnittler, M.; Schulz, W.; Riemay, K.-H.; Krieglsteiner, L. (2007). Checklist of slime moulds (Myxomycetes) of Thuringia. Zeitschrift für Mykologie - Journal of the German Mycological Society, 73(1), 111–136
308. Murr, J. (1918). Zur Pilzflora von Vorarlberg und Liechtenstein II. Oesterreichische botanische Zeitschrift, 67(10–12), 345–356
309. Murr, J. (1922-07). Zur Pilzflora von Vorarlberg und Liechtenstein. III. Österreichische botanische Zeitschrift, 71, 220–223
310. Namysłowska, A. (1938). Śluzowce zebrane w okolicach Stryja przez profesora dra Edwarda Lubicz-Niezabitowskiego / Les myxomycètes recoltés dans les environs de Stryj (Sous-Carpates Orientales) par le Prof. Dr Edouard Lubicz-Niezabitowski. Sprawozdanie Komisyi Fizyograficznej, 72, 453–462
311. Neubert, H. (1980). Myxomyceten aus der Bundesrepublik Deutschland - I Ein neuer Myxomycet aus dem nördlichen Schwarzwald. Zeitschrift für Mykologie, 46(2), 217–220
312. Neubert, H.; Baumann, K. (1986). Myxomyceten aus der Bundesrepublik Deutschland, III. Liste der bislang bekannten Arten. Carolinea, 44, 61–66
313. Neubert, H.; Nowotny, W.; Baumann, K. (1989). Myxomyceten aus der Bundesrepublik Deutschland V (Mit Berücksichtigung von Vorkommen in Oberösterreich). Carolinea, 47, 25–46
314. Neubert, H.; Nowotny, W.; Baumann, K. (1992). Myxomyceten aus Deutschland VIII. (Mit Berücksichtigung von Vorkommen in Oberösterreich). Carolinea - Beiträge zur naturkundlichen Forschung in Südwestdeutschland, 50, 27–44
315. Nixdorf, J. (2022). Myxomyceten in der Naturwaldzelle "Rungstock" bei Olbernhau. Sächsische floristische Mitteilungen, 24, 86–96
316. Notov, A. A.; Gimel'brant, D. E.; Stepanchikova, I. S.; Volkov, V. P. (2022-07-07). Additions to the lichen flora of the Central Forest state natural biosphere reserve. Herald of Tver State University. Series: Biology and Ecology, 2(66), 122–132
317. Novozhilov, Y. K.; Schnittler, M.; Zemlianskaia, I. V. (2005). Synecology of myxomycetes in desert of the northwestern Caspian Lowland. Mikologiya i fitopatologiya, 39(4), 40–52
318. Novozhilov, Y. K.; Zemlianskaia, I. V.; Schnittler, M. (2005). Myxomycetes of the northwestern Caspian deserts. Novosti sistematiki nizhshikh rastenii, 38, 164–170
319. Novozhilov, Y.K., Okun, M.V., Erastova, D.A., Shchepin, O.N., Zemlyanskaya, I.V., García‑Carvajal, E. and Schnittler, M. (2013) Description, culture and phylogenetic position of a new xerotolerant species of Physarum. Mycologia, 105(6), 1535–1546
320. Novozhilov, Y.K., Schnittler, M., Erastova, D.A., Okun, M.V., Schepin, O.N. and Heinrich, E., 2013. Diversity of nivicolous myxomycetes of the Teberda State Biosphere Reserve (Northwestern Caucasus, Russia). Fungal Diversity, 59, pp.109–130
321. Novozhilov, Y.K.; Lebedev, A.N. (2006). Annotated check-list of lignicolous myxomycetes of the Tver Oblast. Mikologiya i Fitopatologiya, 40(3), 236–245
322. Novozhilov, Yu. K. (1986). Нивальные миксомицеты Ленинградской области. Novosti sistematiki nizshikh rastenii, 23, 146–149
323. Novozhilov, Yu. K.; Zemlianskaia, I. V.; Schnittler, M.; Stephenson, S. L. (2006). Myxomycete diversity and ecology in the arid regions of the Lower Volga River Basin (Russia). Fungal Diversity, 23, 193–241
324. Novozhilov, Yu.K., Zemlianskaia, I.V., Schnittler, M. and Fefelov, K.A. (2003) ‘An annotated checklist of the myxomycetes of the northwestern Caspian lowland’, Mycology and Phytopathology, 37(6), pp. 53–65
325. Novozhilov, Yuri K.; Shchepin, Oleg N.; Gmoshinskiy, Vladimir I.; Schnittler, Martin (2020). Myxomycetes of boreal forests of the Laplandskiy State Nature Biosphere Reserve (Kola Peninsula, Russia). Karstenia, 58(2), 292–315
326. Nowotny, W. (1983) ‘Beiträge zur Kenntnis der Myxomyceten Oberösterreichs. I. Die Gattungen Trichia, Hemitrichia und Metatrichia’, Linzer biologische Beiträge, 14(2), pp. 111–126
327. Nowotny, W. (1986). Beiträge zur Kenntnis der Myxomyceten Oberösterreichs II. I. Ergänzungen zu den Gattungen Trichia, Hemitrichia und Metatrichia. Linzer biologische Beiträge, 18(1), 177–189
328. Nowotny, W. (1987). Beiträge zur Kenntnis der Myxomyceten Oberösterreichs III. Linzer biologische Beiträge, 19(2), 273–294
329. Nowotny, W. (1989-06-30). Beiträge zur Kenntnis der Myxomyceten Oberösterreichs IV. Linzer biologische Beiträge, 21(1), 229–245
330. Nowotny, W. (1990). Beiträge zur Kenntnis der Myxomyceten Oberösterreichs V. Linzer biologische Beiträge, 22(1), 97–142
331. Nowotny, W. (1991). Beiträge zur Kenntnis der Myxomyceten Oberösterreichs VI. Linzer biologische Beiträge, 23(1), 79–128
332. Nowotny, W. (1992-07-17). Beiträge zur Kenntnis der Myxomyceten Oberösterreichs VII. Linzer biologische Beiträge, 24(1), 151–206
333. Ohryzek, J. (2021). Observation record: Salaš u Velehradu, Czech Republic
334. Pagitz, K.; Huemer, P. (2018). Tag der Artenvielfalt 2018 – Tirol/Stubaital. Wissenschaftliches Jahrbuch der Tiroler Landesmuseen, 11, 12–45
335. Pagitz, K.; Knoflach, B.; Jedinger, A. (2006-12). GEO-Tag der Artenvielfalt 2006 in Tirol – Erhebungen im Kaisergebirge und an der Schwemm. Berichte des naturwissenschaftlich-medizinischen Vereins in Innsbruck, 93, 169–255
336. Panek, E. & Romański, M. (2010) Śluzowce Myxomycetes. In: Krzysztofiak, L. (ed.) Śluzowce Myxomycetes, grzyby Fungi i mszaki Bryophyta Wigierskiego Parku Narodowego. Suwałki: Stowarzyszenie „Człowiek i Przyroda”, pp. 9–84
337. Pärtel, K.; Suija, A.; Yatsiuk, I. (2021). The Estonian Mycological Collections of Heinrich August Dietrich (1820–1897). Acta Baltica Historiae et Philosophiae Scientiarum, 9(2), 48–78
338. Paul, W.; Janik, P.; Ronikier, A. (2024). Checklist of Myxomycetes (Amoebozoa) of the Polish Tatra Mts. Acta Mycologica, 58, 1–13
339. Pawłowicz, T.; Żebrowski, I.; Micewicz, G. M.; Puchlik, M.; Wilamowski, K.; Sztabkowski, K.; Oszako, T. (2025). First Assessment of the Biodiversity of True Slime Molds in Swamp Forest Stands of the Knyszyn Forest (Northeast Poland) Using the Moist Chambers Detection Method. Forests, 16(8), 1259
340. Pidoplychka, M. M. (1932). Critical materials for the flora of myxomycetes of Ukraine. The Journal of the Biobotanical Cycle of the Academy of Sciences of the Ukrainian SSR, 3-4, 69-102
341. Piltaver, A. (2011). POROČILO O DELU SKUPINE ZA GLIVE
342. Piltaver, A. (2014). Report of the Group for Fungi
343. Piltaver, A. (2015). Poročilo o delu skupine za glive. In: Kljun, I. (ed.), Raziskovalni tabor študentov biologije Slovenske gorice – Sveti Jurij ob Ščavnici 2011. Društvo študentov biologije, Ljubljana
344. Pliszko, A.; Bochynek, A. (2017-08-30). A new record of Badhamia versicolor Lister (Physaraceae) in Poland. Biodiversity Research and Conservation, 45, 23–25
345. Poelt, J. (1956). Schleimpilze aus Südbayern und Tirol. Berichte der Bayerischen Botanischen Gesellschaft zur Erforschung der Flora, 31, 69–75
346. Pouska, V. (2021). Data_SG_hlenky. – Položky z nálezové databáze
347. Prongué, J.-P.; Wiederin, R. (1982). Mykologische Notizen aus dem Ruggeller Riet (FL) (Teil I). Berichte der Botanisch-Zoologischen Gesellschaft Liechtenstein-Sargans-Werdenberg, 11, 45–52
348. Prongué, J.-P.; Wiederin, R. (1990). Die Pilze des Ruggeller Rietes. Berichte der Botanisch–Zoologischen Gesellschaft Liechtenstein–Sargans–Werdenberg, 18, 113–139
349. Pušová, T., 2021. Literární rešerše hlenek v České republice. Bakalářská práce. Česká zemědělská univerzita v Praze, Fakulta životního prostředí
350. Puusepp, V. (1960-05). Limaseentest. Eesti Loodus, (3), 336–338
351. Rachůnková, J. (2014). Hlenky (Myxomycota) v okolí Vaňovského vodopádu v Českém středohoří a v okolí obce Hartvíkovice v okrese Třebíč. Bachelor's thesis, University of Jan Evangelista Purkyně in Ústí nad Labem, Faculty of Science
352. Révay, Á.; Nagy, L. (2005). Myxomycetes data from the Danube–Tisza Interfluve and some other parts of Hungary. Studia bot. hung., 36, 117–121
353. Rist, O.; Schuh, R.; Türk, R.; Zechmeister, H. (2018). Natur in Breitenfurt – Ergebnisse zum Tag der Artenvielfalt 2015. Biosphärenpark Wienerwald Management GmbH, Tullnerbach
354. Ronikier A.; Bochynek A.; Chachuła P.; Kozik J.; Kubiak D.; Perz P.; Salamaga A. (2017). Revision of the genus Licea (Myxomycetes) in Poland. Nova Hedwigia, 104(1–2), 243–272
355. Ronikier, A.; Janik, P. (2020). Trichia sordida (Trichiaceae) – a cryophilous myxomycete found in the Tatra Mountains (Poland), new for Poland and the Carpathians. Fragmenta Floristica et Geobotanica Polonica, 27
356. Ronikier, A.; Lado, C.; Meyer, M.; Wrigley de Basanta, D. (2010). Two new species of nivicolous Lamproderma (Myxomycetes) from the mountains of Europe and America. Mycologia, 102(3), 718–728
357. Ronikier, A.; Perz, P.; Chachuła, P. (2013). First records of Arcyria marginoundulata Nann.-Bremek. & Y. Yamam. (Myxomycetes) in Poland. Acta Mycologica, 48(2), 279–285
358. Ronikier, A.; Ronikier, M. (2007-11-27). New records of nivicolous myxomycetes from the South-Eastern European mountains. Mycologia Balcanica, 4, 143–146
359. Ronikier, A.; Ronikier, M.; Drozdowicz, A. (2008). Diversity of nivicolous myxomycetes in the Gorce mountains – a low-altitude massif of the western Carpathians. Mycotaxon, 103, 337–352
360. Roth, J. (2011). Inventarizační mykologický průzkum na území NPR Úhošť u Kadaně: závěrečná zpráva. Chomutov: AOPK ČR. Manuscript
361. Roth, J. (2013). Inventarizační mykologický průzkum na území PR Čabel – závěrečná zpráva
362. Rücker, T. (1990) ‘Die Pilzflora der Gaisbergwälder bei Salzburg, Österreich’, Verhandlungen der Zoologisch-Botanischen Gesellschaft in Österreich, 127, pp. 165–183
363. Rücker, T. (1997) ‘Die Pilzflora der Stadt Salzburg’, Mitteilungen der Gesellschaft für Salzburger Landeskunde, 137, pp. 325–420
364. Rudolf, K.; Pál-Fám, F.; Morschhauser, T. (2008). A Cserehát nagygombái. Mikológiai Közlemények, Clusiana, 47(1), 45–74
365. Růžičková, J. (1995). Myxomycetes v přirozených porostech jižních Čech. Bachelor's thesis, Jihočeská univerzita v Českých Budějovicích, Biologická fakulta
366. Sadykov, R. E. (2021). Myxomycetes of Kazan forest parks in mid-spring phenological conditions. Geographical research of territorial systems, 11–15
367. Sadykov, R. E.; Potapov, K. O.; Lukyanova, Y. A.; Sadykova, Y. R. (2023). Species Diversity of Myxomycetes of the “Nizhnyaya Kama” National Park and Adjacent Territories in the Early Autumn Period. Mycology and Phytopathology, 57(6), 451–455
368. Salamaga, A. (2013-12-01). Oligonema flavidum (Myxomycetes): a species new to Poland. Polish Botanical Journal, 58(2), 747–749
369. Salamaga, A., Grzesiak, B., Wolski, G.J., Kochanowska, M. and Kochanowski, J. (2016) Preliminary investigations into the slime moulds (Myxogastria) in the "Bory Tucholskie" National Park. Acta Mycologica, 51(1), Article 1077
370. Salamaga, A.; Grzesiak, B.; Kochanowska, M.; Kochanowski, J. (2014). Symphytocarpus trechisporus (Myxogastrea) in Poland. Polish Botanical Journal, 59(2), 279–283
371. Salamaga, A.M. (2021) The Myxobiota of the Łagiewnicki Forest in Łódź (Central Poland). Acta Mycologica, 56, 561
372. Sarycheva, L. A. (1999). Fungi and myxomycetes of the Galich’ya Gora Nature Reserve. Voronezh State University. 150 pp
373. Sarycheva, L. A.; Svetasheva, T. Y.; Bulgakov, T. S.; Popov, E. S.; Malysheva, V. F. (2009-01). Mycobiota of Lipetsk Region [in Russian: Микобиота Липецкой области]. Voronezh State University. ISBN 978-5-9273-1593-0
374. Sarzhevskyi, S. V. (2021). Доповнення до видового складу міксоміцетів Національного природного парку «Святі Гори». Biodiversity, ecology and experimental biology, 23(1), Article 02
375. Schinner, F. (1982) ‘Myxomycetes des Großglockner Gebietes (Hohe Tauern, Österreich): Eine ökologische Studie’, Zeitschrift für Mykologie, 48(1), pp. 165–170
376. Schinz, H. (1913). Floristik und Fortschritte. Myxogasteres. Berichte der Schweizerischen Botanischen Gesellschaft = Bulletin de la Société Botanique Suisse, 22, 1–2
377. Schinz, H. (1932). Fortschritte der Floristik: Plasmodiophorales und Myxogasteres. Berichte der Schweizerischen Botanischen Gesellschaft = Bulletin de la Société Botanique Suisse, 41(1), 96–98
378. Schirmer, P.; Krieglsteiner, L.; Flatau, L. (2015). Revision der Arten der Trichia botrytis-Gruppe mit besonderer Berücksichtigung von Trichia subfusca Rex. Zeitschrift für Mykologie, 81(2), 431–450
379. Schmidt, M. & Täglich, U., 2023. Rote Liste und Gesamtartenliste der Schleimpilze (Myxomycetes inkl. Ceratiomyxomycetes) von Berlin. Berlin: Der Landesbeauftragte für Naturschutz und Landschaftspflege / Senatsverwaltung für Mobilität, Verkehr, Klimaschutz und Umwelt. 34 S
380. Schmitt, J. A. (2002). Ergänzungen zur Pilzflora des Saarlandes – Bereits bekannte, für das Saarland neue Arten, Varietäten und Formen. Teil 1. Abhandlungen der Delattinia, 28, 157–238
381. Schmitt, J. A. (2007). Checklist and Red List of the fungi (Fungi) in the Saarland region, 2nd edition. Abhandlungen der Delattinia, 33, 189–379
382. Schmitt, J. A. (2020). Gesamtartenliste der Pilze (Fungi) des Saarlandes. Rote Liste gefährdeter Pflanzen und Tiere des Saarlandes, Ministerium für Umwelt und Verbraucherschutz und DELATTINIA
383. Schnittler, M. (1998). Nivicole Myxomyceten im Thüringer Wald. Boletus, 22(1), 45–48
384. Schnittler, M. (1999). Blockhalden als Lebensraum für Myxomyceten. Decheniana – Beihefte, 37, 105–109
385. Schnittler, M. and Novozhilov, Y.K., 1996. The myxomycetes of boreal woodlands in Russian northern Karelia: a preliminary report. Karstenia, 36(1), pp. 19–40
386. Schnittler, M., Unterseher, M., Pfeiffer, T., Novozhilov, Y.K. & Fiore-Donno, A.M., 2010. Ecology of sandstone ravine myxomycetes from Saxonian Switzerland (Germany). Nova Hedwigia, 90(3–4), pp.227–302
387. Schnittler, M.; Erastova, D. A.; Shchepin, O. N.; Heinrich, E.; Novozhilov, Y. K. (2015). Four years in the Caucasus – observations on the ecology of nivicolous myxomycetes. Fungal Ecology, 14, 105–115
388. Schnittler, M.; Novozhilov, Y. K. (1998). Late-autumn Myxomycetes of the Northern Ammergauer Alps. Nova Hedwigia, 66(1–2), 205–222
389. Schnittler, M.; Unterseher, M.; Tesmer, J. (2006-03-01). Species richness and ecological characterization of myxomycetes and myxomycete-like organisms in the canopy of a temperate deciduous forest. Mycologia, 98(2), 223–232
390. Scholler, M.; Bernauer, T.; Ebel, C.; Miggel, B.; Murmann-Kristen, L.; Schnittler, M. (2013-12-16). Eine mykologische Bestandsaufnahme des Bannwalds „Wilder See – Hornisgrinde“ (Nordschwarzwald, Baden-Württemberg). Carolinea, 71, 153–159
391. Schreier (1959). Ein Schleimpilz (Brefeldia maxima (Fr.) Rostafinski) mit abnorm entwickeltem Äthalium. Schweizerische Zeitschrift für Pilzkunde, 37(4), 61–63
392. Schubert, M. (1993) ‘Myxomyceten aus Mecklenburg-Vorpommern’, Zeitschrift für Mykologie, 59(2), pp. 223–231
393. Šeniglová, R. (2004). Hlenky (Myxomycetes) v Národním parku Podyjí / Slime Molds (Myxomycetes) in the Podyjí National Park. Thayensia, 6, 21–29
394. Shchepin, O. N. (2021). Скрытое разнообразие темноспоровых миксомицетов (Myxomycetes): таксономический и экологический аспекты. Candidate of Biological Sciences thesis, Komarov Botanical Institute, Russian Academy of Sciences, Saint Petersburg
395. Shchepin, O. N.; López Villalba, Á.; Inoue, M.; Prikhodko, I. S.; Erastova, D. A.; Okun, M. V.; Woyzichovski, J.; Yajima, Y.; Gmoshinskiy, V. I.; Moreno, G.; Novozhilov, Y. K.; Schnittler, M. (2024-02-10). DNA barcodes reliably differentiate between nivicolous species of Diderma (Myxomycetes, Amoebozoa) and reveal regional differences within Eurasia. Protist, 175(2), 126023
396. Shchepin, O. N.; Novozhilov, Y. K.; Schnittler, M. (2016). Disentangling the taxonomic structure of the Lepidoderma chailletii-carestianum species complex (Myxogastria, Amoebozoa): genetic and morphological aspects. Protistology, 10(4), 117–129
397. Shchepin, O. N.; Schnittler, M.; Erastova, D. A.; Prikhodko, I. S.; Borg Dahl, M.; Azarov, D. V.; Chernyaeva, E. N.; Novozhilov, Y. K. (2019). Community of dark-spored myxomycetes in ground litter and soil of taiga forest (Nizhne-Svirskiy Reserve, Russia) revealed by DNA metabarcoding. Fungal Ecology, 39, 80–93
398. Shchepin, O.; Novozhilov, Y.; Woyzichovski, J.; Bog, M.; Prikhodko, I.; Fedorova, N.; Gmoshinskiy, V.; Borg Dahl, M.; Dagamac, N.H.A.; Yajima, Y.; Schnittler, M. (2022-01). Genetic structure of the protist Physarum albescens (Amoebozoa) revealed by multiple markers and genotyping by sequencing. Molecular Ecology, 31(1), 372–390
399. Shirokikh, A. A.; Shirokikh, I. G. (2018-11). Обнаружение миксогастриевых миксомицетов в парках города Кирова. Mycology and Algology in Russia. XX–XXI Century: Shift of Paradigms (Conference Program), Moscow State University
400. Singer, H., Moreno, G. and Illana, C. (2001) ‘Nivicolous Myxomycetes from Tyrol (Austria). II. The genus Lamproderma’, Österreichische Zeitschrift für Pilzkunde, 10, pp. 25–42
401. Singer, H.; Moreno, G.; Illana, C.; Sánchez, A. (2003). Lamproderma retirugisporum spec. nova, a misinterpreted species of the Myxomycetes. Österreichische Zeitschrift für Pilzkunde, 12, 13–21
402. Skvortsova, A. V. (2017). Новые данные о миксомицетах природного парка «Щербаковский»
403. Soldatenkova, A., Baranova, Y., Alexandrova, A., Matveev, A., Gmoshinskiy, V. & Vlasenko, A. (2020) New data on Myxomycetes of North‑Eastern Russia. BIO Web of Conferences, 24, 00084
404. Spáčilová, J. (1996). Mykologický průzkum NPR Mionší. Ms., Správa CHKO Beskydy, Rožnov pod Radhoštěm
405. Štěpka, J. (2017). Hlenky (Myxomycetes) v okolí obce Zubrnice v Českém středohoří. Bachelor's thesis, Univerzita Jana Evangelisty Purkyně v Ústí nad Labem
406. Štěpka, J. (2020). Hlenky (Myxomycetes) na vybraných lokalitách v Českém středohoří a okolí. Diploma thesis, Univerzita Jana Evangelisty Purkyně v Ústí nad Labem, Přírodovědecká fakulta
407. Stojakowska, W. (1983) ‘Myxomycetes of the Sudetes’, Acta Mycologica, 19(2), pp. 207–243
408. Stojanowska, W. (1977) Slime mold flora of the Ślęża massif. Acta Mycologica, 13(2), 245–256
409. Stojanowska, W. (1977). Changes in slime mould flora in the reservation of Puszczy Śnieżnej Białki. Acta Mycologica, 13(1), 99–107
410. Stojanowska, W. (1980). Comparison of Myxomycetes of the forest in Skarszyn and of the beech reserve in Muszkowice. Acta Mycologica, 16(2), 221–230
411. Stojanowska, W. (1981). Myxomycetes of the rotting cherry wood. Acta Mycologica, 17(1–2), 125–129
412. Stojanowska, W. (1984) Śluzowce (Myxomycetes) polskich Karkonoszy. Prace Karkonoskiego Towarzystwa Naukowego, 41, 71–90
413. Stojanowska, W. (2004) Rozmieszczenie śluzowców (Myxomycetes) w Karkonoszach. Przyroda Sudetów, 7, 93–108
414. Stojanowska, W. (2004). Śluzowce (Myxomycetes) Przedgórza Sudeckiego na tle śluzowców Sudetów i Pogórza Sudeckiego. Przyroda Sudetów Zachodnich, 7, 63–92
415. Stojanowska, W. and Panek, E. (2002) Changes in the myxomycete biota of the "Łężczok" nature reserve near Racibórz (SW Poland). Acta Mycologica, 37(1–2), 13–28
416. Stojanowska, W. and Panek, E. (2005) Biota śluzowców doliny Pośny w Parku Narodowym Gór Stołowych. Szczeliniec, 9, 75–94
417. Stojanowska, W.; Panek, E. (2003). The genus Craterium (Myxomycetes) in Poland. Acta Mycologica, 38(1–2), 61–69
418. Stojanowska, W.; Panek, E. (2004). Myxomycetes of the nature reserve near Walbrzych (SW Poland). Part II. Dependence on the substrate and seasonality. Acta Mycologica, 39(2), 147–159
419. Strazdiņa, L.; Kluša, J.; Klušs, A.; Leimanis, I.; Opmanis, A. (2019). Krimuldas mežaparka dabas retumi. Latvijas Veģetācija, 29, 59–76
420. Strazdiņa, L.; Kluša, J.; Leimanis, I.; Laime, S.; Birziņa, L.; Oļehnoviča, E.; Opmanis, A. (2021). Additions to checklists of cryptogams in Latvia (records from 2017–2019). Latvijas Veģetācija, 31, 41–60
421. Svrček, M. (1959-07-20). Resultate der mykologischen Durchforschung Böhmens für das Jahr 1958, I. Der Winter und Frühlingsaspekt der mittelböhmischen Mykoflora. Česká Mykologie, 13(3), 153–159
422. Svrček, M. (1970-04-20). Lamproderma sauteri Rost. (New records. 5.). Česká Mykologie, 24(2), 103–104
423. Svrček, M. (1972). Myxomycetes developed in moist chamber cultures. I. Česká Mykologie, 26(2), 103–113
424. Svrček, M. (1976). SPR Boubínský prales, houby
425. Svrček, M. (1984). SPR Březina u Milešova v Českém středohoří – houby. Unpublished report, ÚSOP (AOPK ČR), Praha
426. Svrček, M. (1986). SPR Karlštejn. Lesy mezi Dub bratří a Královskou studánkou. Manuscript, Ústřední seznam ochrany přírody (ÚSOP), Praha
427. Svrček, M. (1986). SPR Kuchyňka (636 m n. m.) v Brdských hřebenech u Hostomic pod Brdy. Manuscript, Ústřední seznam ochrany přírody (ÚSOP), Praha
428. Svrček, M. (1987). Mykofyta zjištěná na úz. SPR Prokopské údolí. Manuscript, Ústřední seznam ochrany přírody (ÚSOP), Praha
429. Svrček, M. (1990). SPR Kokořínský důl. Mykoflora 1987-90
430. Svrček, M. (1990). Zpráva o mykologickém průzkumu SPR Kuchyňka (636 m n. m.) v Brdských hřebenech u Hostomic p. Brdy
431. Svrček, M. (1990-10-22). A report on mycological trips to Krkonoše Mts., Bohemia, in the years 1986–1989. II. Czech Mycology, 44(3), 140–146
432. Svrček, M. (1992). Zpráva o mykologickém inventarizačním průzkumu SPR Podbabské skály. Manuscript, AOPK ČR, Prague
433. Svrček, M. (1993). Divoká Šárka houby. Manuscript, deposited at Ústřední seznam ochrany přírody (ÚSOP), Agentura ochrany přírody a krajiny ČR, Prague
434. Svrček, M. (1994). Výsledky mykologického inventarizačního průzkumu SPR Divoká Šárka v roce 1994. Inventarizační průzkum. AOPK ČR (ÚSOP)
435. Svrček, M.; Kubička, J. (1964-07-14). Fungi from the Žofínský Virgin Forest in the Novohradské mountains (Southern Bohemia). Česká Mykologie, 18(3), 157–179
436. Svrček, M.; Kubička, J. (1971). Zweiter Beitrag zur Kenntnis der Mykoflora des Urwaldes „Žofínský prales“ im Gebirge Novohradské hory (Südböhmen). Česká Mykologie, 25(2), 103–111
437. Ślusarczyk, D. (2010). Some observations of slime moulds on wood and litter in beech forests. Acta Mycologica, 45(2), 239–246
438. Ślusarczyk, D. M. (2021-06-04). First Record of Slime Molds in Biebrza National Park (NE Poland). Acta Mycologica, 56, 564
439. Täglich, U. (2020). Drei Kiefernholz besiedelnde Myxomyceten in Sachsen-Anhalt. Boletus - Pilzkundliche Zeitschrift, 41(2), 157–163
440. Tchesunov, A. V.; Kaljakina, N. M.; Bubnova, E. N. (2008). A Catalogue of Biota of the White Sea Biological Station of the Moscow State University. KMK Scientific Press Ltd., Moscow, 384 pp
441. Tesmer, J., Rulik, B., Spiegel, F.W., Shadwick, J. and Schnittler, M., 2005. Protostelids from German Beech forests. Mycological Progress, 4, pp.267–271
442. Tóth, S. (1954). Adatok Magyarország mikroszkopikus gombáinak ismeretéhez. I. Botanikai Közlemények, 45(3–4), 241–246
443. Tóth, S. (1991). Adatok az Alpokalja mikroszkopikus gombáinak ismeretéhez II. Savaria – A Vas Megyei Múzeumok Értesítője, 20(2), 217–232
444. Tóth, S. (1994) Microscopic fungi of the Pilis and Visegrád Mts, Hungary. Studia Botanica Hungarica, 25, 21–57
445. Tsurykau, A. (2017). Licea parasitica (Myxomycetes) new to Belarus. Botanica Lithuanica, 23(1), 63–64
446. Uvarova, M. A.; Smelova, D. V.; Khizhnyakova, A. S. (2019). К вопросу о биоте миксомицетов национального парка «Смольный». Mordovskiy zapovednik, 16, 3–5
447. Valter, J. (2001). Houby přírodní rezervace Borkovická blata. Manuscript, deposited in: Okresní úřad Tábor; J. Valter, Tábor; F. Kotlaba, Praha
448. Valtz, Y.; Rishavi, L. (1872). List of the collection of myxomycetes and fungi collected by A.S. Rogovich, Ya. Ya. Valtz and L. Rishavi. Notes of the Kyiv Society of Naturalists, 2(2), 187–189
449. Vass, A. (1962). Újabb adatok a Mecsek-hegység mikroszkopikus gombaflórájához. II. Janus Pannonius Múzeum Évkönyve (1961), 51–57
450. Vass, A. (1972) Adatok a Zselicség mikrogomba flórájának ismeretéhez. Janus Pannonius Múzeum Évkönyve, 16 (1971), 7–12
451. Vass, A. (1975). Újabb adatok a Mecsek-hegység és környékének mikroszkopikus gombaflórájához V. Janus Pannonius Múzeum Évkönyve, 17–18, 7–14
452. Vass, A. (1976). Újabb adatok a Mecsek hegység és környéke mikroszkopikus gombaflórájához VII
453. Vass, A. (1978). A Barcsi Ősborókás mikroszkopikus gombái I. Dunántúli Dolgozatok, Természettudományi Sorozat, 1, 37–43
454. Vass, A. (1981). Újabb adatok a Mecsek hegység és környékének mikroszkopikus gombaflórájához IX. Janus Pannonius Múzeum Évkönyve, 25, 21–26
455. Vass, A. (1983). Recent data on the microscopic fungi flora of the Mecsek Mountains and environs XI. Janus Pannonius Múzeum Évkönyve, 27, 7–13
456. Vass, A. (1984). Recent data on the microscopic fungi of the Mecsek Mountains and environs XII. Janus Pannonius Múzeum Évkönyve, 28, 1-10
457. Vass, A. (1989). The microscopic fungi of the Old Juniper Woodland of Barcs, South Hungary. Janus Pannonius Múzeum Évkönyve, 33 (1988), 7–17
458. Vass, A.; Horvatovich, S. (1984). Myxomycetes
459. Vass, A.; Tóth, S. (1959). Mikroszkopikus gombák a Mecsek-hegységből. II. Janus Pannonius Múzeum Évkönyve, 1959, 45–54
460. Vimba, E. & Adamonytė, G., 2003. Additional data on Latvian myxomycetes. Folia Cryptogamica Estonica, 40, 57–61
461. Viunnyk, V. O.; Leontyev, D. V.; López-Villalba, Á. (2023). First records of bryophilous myxomycetes in the lowlands of Ukraine reveal an undescribed species of Lamproderma. Czech Mycology, 75(2), 191–206
462. Viunnyk, V.O.; Leontyev, D.V. (2020). Biodiversity and ecology of myxomycetes of the botanical reserve of local importance "Sharivskyi" (Kharkiv region, Ukraine). Biodiversity, Ecology and Experimental Biology, 22(2), 24–29
463. Vlasenko, A. V. (2011). Myxomycetes of the Tigirek State Nature Reserve (an annotated check-list). Trudy Tigirekskogo Zapovednika, 4, 54–56
464. Vlasenko, A.; Shanmak, R.; Sambyla, C. (2021). First data on Myxomycetes of the State Nature Preserve "Sut-Khol", Republic of Tuva (Tyva), Russia. BIO Web of Conferences, 38, 00136
465. Vodlozersky National Park (2022). Грибы и слизевики Национального парка «Водлозерский»
466. von Moesz, G. (1930). Pilze aus der Umgebung des Balaton und des Bakony-Gebirges. Arbeiten der I. Abt. des Ungarischen Biologischen Forschungsinstitutes, Tihany, 88–119
467. Vondrová, S. (1991). Echinostelium minutum and other Myxomycetes developed in moist chamber culture. Česká Mykologie, 45(1-2), 27–32
468. Wagner, S.; Mrkvicka, A. (2021). Natur im Lainzer Tiergarten: Ergebnisse zum Tag der Artenvielfalt 2016 und 2018. Biosphärenpark Wienerwald Management GmbH
469. Wagner, S.; Wrbka, E. (2020). Natur in Alland – Ergebnisse zum Tag der Artenvielfalt 2017. Biosphärenpark Wienerwald Management GmbH
470. Wichanský, E. (1958-10-20). Myxomyces Diderma spumarioides Fr. in Prague. Česká Mykologie, 12(4), 218–219
471. Wichanský, E. (1959-10-20). De speciebus generis Lycogala in Bohemia. Česká Mykologie, 13(4), 223–226
472. Wichanský, E. (1962-01). Několik druhů, odrůd a forem vzácnějších nebo méně známých hlenek (Myxomycetes) z nálezů v letech 1957 až 1960 v ČSSR. Česká Mykologie, 16(1), 34–43
473. Wichanský, E. (1962-04-13). Badhamia affinis Rost. Česká Mykologie, 16(2), 116
474. Wichanský, E. (1963-04-09). Československé druhy rodu Trichia — závitěnka (Myxomycetes). Česká Mykologie, 17(2), 91–97
475. Wichanský, E. (1964-01-25). Myxomycetum species rariores vel minus cognitae in Bohemia et Moravia (= Vzácnější a méně známé druhy hlenek v Čechách a na Moravě). Česká Mykologie, 18(1), 55–59
476. Wichanský, E. (1964-10). Didymium macrospermum Rost. – dvoukožnatka velkovýtrusá. Česká Mykologie, 18(4), 236–237
477. Wichanský, E. (1966). Physarum rubiginosum Fr. Česká Mykologie, 20(1), 61
478. Wichanský, E. (1966). The Czechoslovakian species of the genus Hemitricha (Myxomycetes). Česká Mykologie, 20(3), 189–198
479. Wichanský, E. (1968) The Czechoslovakian species of the genus Arcyria (Myxomycetes). Česká Mykologie, 22(2), 129–145
480. Wieser, C.; Komposch, C.; Krainer, K.; Wagner, J. (2004). 6. GEO-Tag der Artenvielfalt Griffner Schlossberg und Griffner See, Kärnten 11./12. Juni 2004. Carinthia II, 194(114), 537–590
481. Wilga, M. S.; Ciechanowski, M. (2007). Ostoja grzybów wielkoowocnikowych i śluzowców w Lasach Oliwskich (Trójmiejski Park Krajobrazowy). Chrońmy Przyrodę Ojczystą, 63(6), 82–101
482. Wrońska, B. (1974) Materiały do znajomości śluzowców (Myxomycetes) Lubelszczyzny. Annales Universitatis Mariae Curie‑Skłodowska, Sectio C, Biologia, 29, 471–476
483. Yatsiuk, I. I.; Leontyev, D. V.; Shlakhter, M. L. (2017). Myxomycetes of National Nature Park Slobozhanskiy (Ukraine): biodiversity and noteworthy species. Nordic Journal of Botany, 36(1–2), e01605
484. Yatsiuk, I., Leontyev, D., Schnittler, M., Ehlers, T., Mikryukov, V. and Kõljalg, U. (2025) Arcyria and allied genera: taxonomic backbone and character evolution. Fungal Systematics and Evolution, 15, pp. 97–118
485. Yatsiuk, I.; Kastanje, V.; Adamonyte, G. (2020). Myxomycetes of Estonia - unpublished specimens. (Dataset)
486. Yatsiuk, I.; Leontyev, D. (2020-03-26). Two species of nivicolous myxomycetes that formed fruiting bodies during three spring seasons in the lowlands of the Eastern Ukraine. Phytotaxa, 437(3), 147–155
487. Yatsyna, A. P.; Moroz, E. L. (2023). Lichens and myxomycetes of floodplain oak forests of the reserve «Lipichanskaya Pushcha» (Grodno Region, Belarus). Diversity of plant world, 1(16), 25–35
488. Yatsyna, A. P.; Moroz, E. L. (2023). Lichens and myxomycetes of oak forests of the planned reserve «Ross-Neman» (Grodno Region, Belarus). Diversity of Plant World, 4(19), 36–44
489. Yatsyna, A. P.; Moroz, E. L. (2024). Lichens and myxomycetes of oak forests of the National Park Belovezhskaya Pushcha (Belarus). Diversity of plant world, 2(21), 24–37
490. Záhorovská, E. (1989). Príspevok k poznaniu slizoviek (Myxomycetes) Slovenska. In VIII. celostátní vědecká mykologická konference: sborník referátů a souhrnů referátů (Brno, 28.VIII.–1.IX.1989), 59–60. Vysoká škola zemědělská v Brně
491. Záhorovská, E. (1990). Pravé slizovky Devínskej Kobyly. Acta Facultatis rerum naturalium Universitatis Comenianae, Botanica, 38, 23–33
492. Záhorovská, E. (1992). Pravé slizovky (Myxomycetes) okolia Zvolena. Acta Facultatis rerum naturalium Universitatis Comenianae, Botanica, 39, 3–12
493. Záhorovská, E. (1994). Slizovky (Myxomycetes) vypestované vo vlhkých komôrkach. Mykologické listy, 52, 7–12
494. Záhorovská, E. (1996). Slizovky (Myxomycetes) Slovenska. Mykologické Listy, 57, 13–18
495. Záhorovská, E.; Lisická, E. (2002). Lamproderma arcyrioides (Myxomycota, Stemonitidaceae) fruktifikujúca na lišajníkoch. Mykologické listy, 80, 12–13
496. Záhorovská, E.; Lišková, D.; Vozárová, M. (1996). Mykoflóra ostrova Sihoť a Slovanského ostrova. Spravodajca slovenských mykológov, 14, 21–23
497. Zelle, M. (1921) Materials for the myxomycete flora of Ukraine. Bulletin of the Kyiv Botanical Garden, 2, 31–39
498. Zeller, L. and Tóth, S., 1976/1977. Myxomycetes data from Hungary II. Annales Universitatis Scientiarum Budapestiensis de Rolando Eötvös Nominatae, Sectio Biologica, 18/19, pp. 137–154
499. Zeller, L.; Tóth, S. (1960). Mikroskopische Pilze aus dem Bükk-Gebirge. Botanikai Közlemények, 48(3–4), 228–231
500. Zemlianskaia, I. V.; Novozhilov, Yu. K. (2010). Myxomycetes from the salt-domes near Elton Lake. Mikologiya i Fitopatologiya, 44(6), 516–523
501. Zemlyanskaya, I. V.; Novozhilov, Y. K. (2022). New data on myxomycete diversity of the Republic of Tatarstan (Russia). Novosti sistematiki nizshikh rastenii, 56(1), 71–84
502. Zemlyanskaya, I. V.; Novozhilov, Yu. K. (2022). New data on myxomycetes in the Republic of Kalmykia (Russia). Novosti sistematiki nizshikh rastenii, 56(2), 309–321
503. Zemlyanskaya, I. V.; Rebriev, Yu. A. (2008). Myxomycetes. In: Annotated lists of species of fungi and myxomycetes (Proceedings of the IX Working Meeting of the Commission for the Study of Macromycetes, Vyoshenskaya, 4–10 Oct 2006), 57–60. Rostov-on-Don
504. Zemlyanskaya, I.V., 2003. Myxomycetes of Bogdinsko-Baskunchaksky Nature Reserve. Mikologiya i Fitopatologiya, 37(4), pp.40–47
505. Zemlyanskaya, I.V., Smolnyakova, Yu.A., Kurbatova, M.E. & Novozhilov, Yu.K., 2018. Новые данные о миксомицетах (Myxomycetes) природного парка «Волго‑Ахтубинская пойма». Вестник Воронежского государственного университета. Серия: Химия. Биология. Фармация, 2018(2), 129–134
506. Авторский коллектив исследователей (2014) Летопись природы Висимского государственного природного биосферного заповедника за 2013 год. Академия Естествознания. ISBN 978‑5‑91327‑314‑7
507. Бухтоярова, Н. Ю.; Гмошинский, В. И. (2017). Миксомицеты Южного лесничества Центрально-Лесного государственного природного биосферного заповедника. Конференция «Ломоносов 2017», Секция «Микология и альгология»
508. Бухтоярова, Н. Ю.; Гмошинский, В. И. (2017). Итоги изучения видового разнообразия миксомицетов (кл. Myxomycetes) Южного лесничества Центрально-Лесного государственного биосферного заповедника с 2014 по 2016 год. Вклад заповедной системы в сохранение биоразнообразия и устойчивое развитие, конференция, Заповедный, Тверская область, Россия, 14–17 августа 2017
509. Гмошинский В. И.; Бухтоярова Н. Ю.; Матвеев А. В. (2015). Изучение видового разнообразия миксомицетов (кл. Myxomycetes) Центрально-Лесного государственного природного биосферного заповедника в 2015-2016 г
510. Гмошинский В.И., Дунаев Е.А., Киреева Н.И. Определитель миксомицетов Московского. региона. М.: АРХЭ, 2021. 384 с. 4
511. Гмошинский, В.И. & Бухтоярова, Н.Ю. (2016) Изучение видового разнообразия миксомицетов (кл. Myxomycetes) Южного лесничества Центрально-Лесного государственного природного биосферного заповедника в 2015 году. В: Летопись природы ФГБУ «Центрально-Лесной государственный природный биосферный заповедник» за 2015 год, т. 55, пос. Заповедный, с. 105–162
512. Гмошинский, В.И. & Матвеев, А.В., 2021. Изучение видового разнообразия миксомицетов Центрально‑Лесного биосферного заповедника в 2019 г. In: Динамика явлений и процессов в природном комплексе заповедника. Летопись природы, книга 60. пос. Заповедный: Центрально‑Лесной государственный природный биосферный заповедник, pp. 155–174
513. Горунова А.В.; Эбель М.А. (2017). Представители семейства Stemonitidaceae в биоте миксомицетов «Волго-Ахтубинской поймы»
514. Евстигнеев, О.И. and Федотов, Ю.П., 2007. Флора сосудистых растений заповедника" Брянский лес". Государственный природный биосферный заповедник" Брянский лес"
515. Кривомаз, Т.І. (2004). Міксоміцети Шацького національного природного парку. In: Наукові дослідження 1994–2004: Матеріали міжнародної науково-практичної конференції (Світязь, 17–19 травня 2004)
516. Летопись природы (2008). Книга 15. 2008 год — Myxomycota – Слизевики
517. Луптакова, А. Д. (2019). Миксомицеты Красносамарского лесного массива Самарской области. Конференция «Ломоносов 2019», Секция «Микология и альгология»
518. Мишулин, А.А. (2018) Данные о биоте миксомицетов Владимирской области. В: Материалы Международного молодежного научного форума «Ломоносов‑2018». М.: МАКС Пресс. ISBN 978‑5‑317‑05800‑5
519. Мониторинг и оценка состояния растительного мира: материалы VI Международной научной конференции (Минск–Лясковичи, 9–13 октября 2023). (2023) Минск: ИВЦ Минфина. ISBN 978‑985‑880‑362‑9
520. Мосолов, Н. А. (1906). Грибы. Списокъ грибовъ, найденныхъ въ Подольскомъ уѣздѣ. Москва: Типо-лит. Т-ва И. Н. Кушнеревъ и Ко
521. Мурашкинский, К. Е. (1911). ОПИСАТЕЛЬНЫЙ КАТАЛОГЪ ЕСТЕСТВЕННО-ИСТОРИЧЕСКАГО МУЗЕЯ Нижегородскаго Губернскаго Земства. Руководство къ изученію природы Нижегородскаго края. Выпускъ 3-й. Отдѣлъ ботаническій: каталогъ гербарія грибовъ. Нижний Новгород: Типография В. Ройского и И. Карнева
522. Новожилов Ю.К.; Кошелева А.П. (2025). Миксомицеты (класс Myxomycetes) Оренбургского природного государственного заповедника. Институт степи
523. Смольнякова, Ю.А., Котельникова, Д.А. and Землянская, И.В., 2015. Миксомицеты памятника природы «Чапурниковская балка» Волгоградской области. In Материалы всероссийской научной конференции с международным участием, посвященной 10-летию создания кафедры ботаники и экологии растений и кафедры микробиологии СурГУ. Сургут (p. 99)
524. Смольнякова, Ю.А.; Котельникова, Д.А.; Землянская, И.В. (2015-08-08). Миксомицеты байрачных лесов Волгограда. Материалы VII всероссийской микологической школы-конференции с международным участием «Биотические связи грибов: мосты между царствами», ЗБС МГУ, 216–222
525. Фефелов, К. А. (2010). Базовый список миксомицетов Южно-Уральского государственного заповедника. Труды Тигирекского заповедника
526. Фокшей, С. І.; Держипільський, Л. М. (2019). Нові мікологічні знахідки на території НПП «Гуцульщина». Науковий вісник Чернівецького університету. Біологія (Біологічні системи), 13(2), 191
527. Широких, И. Г.; Широких, А. А. (2010). Разнообразие миксомицетов в лесопарковой зоне г. Кирова. Иммунопатология, аллергология, инфектология, 54–55
528. Шуканов, А. С.; Мороз, Е. Л.; Малиновский, О. Л. (1988). Миксомицеты Нарочано-Вилейской низины. Вестник Белорусского государственного университета имени В. И. Ленина. Серия 2, Химия. Биология. География, (2), 23–25
